# Efficient Discretization of Optimal Transport

**DOI:** 10.3390/e25060839

**Published:** 2023-05-24

**Authors:** Junqi Wang, Pei Wang, Patrick Shafto

**Affiliations:** 1 Department of Math & CS, Rutgers University, Newark, NJ 07102, USA; junqi.wang@rutgers.edu (J.W.); peiwang@rutgers.edu (P.W.); 2 School of Mathematics, Institute for Advanced Study, Princeton, NJ 08540, USA

**Keywords:** optimal transport, entropy regularization, discretization, gradient descent

## Abstract

Obtaining solutions to optimal transportation (OT) problems is typically intractable when marginal spaces are continuous. Recent research has focused on approximating continuous solutions with discretization methods based on i.i.d. sampling, and this has shown convergence as the sample size increases. However, obtaining OT solutions with large sample sizes requires intensive computation effort, which can be prohibitive in practice. In this paper, we propose an algorithm for calculating discretizations with a given number of weighted points for marginal distributions by minimizing the (entropy-regularized) Wasserstein distance and providing bounds on the performance. The results suggest that our plans are comparable to those obtained with much larger numbers of i.i.d. samples and are more efficient than existing alternatives. Moreover, we propose a local, parallelizable version of such discretizations for applications, which we demonstrate by approximating adorable images.

## 1. Introduction

Optimal transport is the problem of finding a coupling of probability distributions that minimizes cost [1], and it is a technique applied across various fields and literatures [2,3]. Although many methods exist for obtaining optimal transference plans for distributions on discrete spaces, computing the plans is not generally possible for continuous spaces [4]. Given the prevalence of continuous spaces in machine learning, this is a significant limitation for theoretical and practical applications.

One strategy for approximating continuous OT plans is based on discrete approximation via sample points. Recent research has provided guarantees on the fidelity of discrete, sample-location-based approximations for continuous OT as the sample size N→∞ [5]. Specifically, by sampling large numbers of points $S_{i}$ from each marginal, one may compute a discrete optimal transference plan on $S_{1}\times S_{2}$, with the cost matrix being derived from the pointwise evaluation of the cost function on $S_{1}\times S_{2}$.

Even in the discrete case, obtaining minimal cost plans is computationally challenging. For example, Sinkhorn scaling, which computes an entropy-regularized approximation for OT plans, has a complexity that scales with $|S_{1}\times S_{2}|$ [6]. Although many comparable methods exist [7], all of them have a complexity that scales with the product of sample sizes, and they require the construction of a cost matrix that also scales with $|S_{1}\times S_{2}|$.

**Figure 1:**
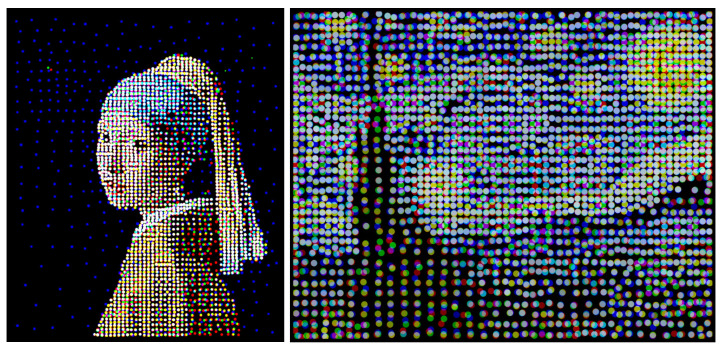
Discretization of “Girl with a Pearl Earring” and “Starry Night” using EDOT with 2000 discretization points for each RGB channel. k=2, $\zeta =0.01\times {\mathrm{diam}}^{2}$.

We have developed methods for optimizing both sampling locations and weights for small N approximations of OT plans (see Figure 1). In Section 2, we formulate the problem of fixed size approximation and reduce it to discretization problems on marginals with theoretical guarantees. In Section 3, the gradient of entropy-regularized Wasserstein distance between a continuous distribution and its discretization is derived. In Section 4, we present a stochastic gradient descent algorithm that is based on the optimization of the locations and weights of the points with empirical demonstrations. Section 5 introduces a parellelizable algorithm via decompositions of the marginal spaces, which reduce the computational complexity by exploiting intrinsic geometry. In Section 6, we analyze time and space complexity. In Section 7, we illustrate the advantage of including weights for sample points by providing a comparison with an existing location that is only based on discretization.

## 2. Efficient Discretizations

Optimal transport (OT): Let $\left(X,d_{X}\right)$, $\left(Y,d_{Y}\right)$ be compact Polish spaces (complete separable metric spaces), μ∈P(X), ν∈P(Y) be probability distributions on their Borel-algebras, and c:X×Y→R be a cost function. Denote the set of all joint probability measures (couplings) on X×Y with marginals μ and ν by Π(μ,ν). For the cost function *c*, the optimal transference plan between μ and ν is defined as in [1]: $\gamma \left(\mu ,\nu \right)\mathrm{argmin}{\;}_{\pi \in \Pi (\mu ,\nu )}\langle c,\pi \rangle$, where $\langle c,\pi \rangle \int_{X\times Y}c\left(x,y\right)d\pi \left(x,y\right)$.

When X=Y, the cost $c\left(x,y\right)=d_{X}^{k}\left(x,y\right)$, $W_{k}\left(\mu ,\nu \right)={\langle c,\gamma \left(\mu ,\nu \right)\rangle }^{1/k}$ defines the *k*-Wasserstein distance between μ and ν for k≥1. Here, $d_{X}^{k}\left(x,y\right)$ is the *k*-th power of the metric $d_{X}$ on *X*.

Entropy regularized optimal transport (EOT)  [5,8] was introduced to estimate OT couplings with reduced computational complexity: $\gamma_{\lambda }\left(\mu ,\nu \right):=\mathrm{argmin}{\;}_{\pi \in \Pi (\mu ,\nu )}\langle c,\pi \rangle +\lambda \mathrm{KL}\left(\pi ||\mu ⊗\nu \right)$, where λ>0 is a regularization parameter, and the regularization term KL(π||μ⊗ν):=$\int \log(\frac{d\pi }{d\mu ⊗d\nu })d\pi$ is the Kullback–Leibler divergence. The EOT objective is smooth and convex, and its unique solution with a given discrete (μ,ν,c) can be obtained using a Sinkhorn iteration (SK)  [9].

However, for large-scale discrete spaces, the computational cost of SK can still be unfeasible [6]. Even worse, to even apply the Sinkhorn iteration, one must know the entire cost matrix over the large-scale spaces, which itself can be a non-trivial computational burden to obtain; in some cases, for example, where the cost is derived from a probability model [10], it may require intractable computations [11,12].

The Framework: We propose the optimization of the location and weights of a fixed size discretization to estimate the continuous OT. The discretization on X×Y is completely determined by those on *X* and *Y* to respect the marginal structure in the OT. Let $m,n\in Z^{*}$, $\mu_{m}\in P\left(X\right)$, $\nu_{n}\in P\left(Y\right)$ be a discrete approximation of μ and ν, respectively, with $\mu_{m}=\sum_{i=1}^{m}w_{i}\delta_{x_{i}}$, $\nu_{n}=\sum_{j=1}^{n}u_{j}\delta_{y_{j}}$, $x_{i}\in X$, $y_{j}\in Y$, and $w_{i},u_{j}\in R^{+}$. Then, the EOT plan $\gamma_{\lambda }\left(\mu ,\nu \right)\in \Pi \left(\mu ,\nu \right)$ for the OT problem (μ,ν,c) can be approximated by the EOT plan $\gamma_{\lambda }\left(\mu_{m},\nu_{n}\right)\in \Pi \left(\mu_{m},\nu_{n}\right)$ for the OT problem $\left(\mu_{m},\nu_{n},c\right)$. There are three distributions that have their discrete counterparts; thus, with a fixed size $m,n\in Z^{*}$, a naive idea about the objective to be optimized can be

(1) $$\begin{array}{c} \Omega_{k,\rho }\left(\mu_{m},\nu_{n}\right)=W_{k}^{k}\left(\mu ,\mu_{m}\right)+W_{k}^{k}\left(\nu ,\nu_{n}\right)+\rho W_{k}^{k}\left(\gamma_{\lambda }\left(\mu ,\nu \right),\gamma_{\lambda }\left(\mu_{m},\nu_{n}\right)\right), \end{array}$$

where $W_{k}^{k}\left(\phi ,\psi \right)$ represents the *k*-th power of *k*-Wasserstein distance between measures ϕ and ψ. The hyperparameter ρ>0 balances between the estimation accuracy over marginals and that of the transference plan, while the weights on marginals are equal.

To properly compute $W_{k}^{k}\left(\gamma_{\lambda }\left(\mu ,\nu \right),\gamma_{\lambda }\left(\mu_{m},\nu_{n}\right)\right)$, a metric $d_{X\times Y}$ on X×Y is needed. We expect $d_{X\times Y}$ on *X*-slices or *Y*-slices to be compatible with $d_{X}$ or $d_{Y}$, respectively; furthermore, we may assume that there exists a constant A>0 such that:

(2) $$\begin{array}{c} \max\left\{d_{X}^{k}\left(x_{1},x_{2}\right),d_{Y}^{k}\left(y_{1},y_{2}\right)\right\}\leq d_{X\times Y}^{k}\left(\left(x_{1},y_{1}\right),\left(x_{2},y_{2}\right)\right)\leq A\left(d_{X}^{k}\left(x_{1},x_{2}\right)+d_{Y}^{k}\left(y_{1},y_{2}\right)\right). \end{array}$$

For instance, (2) holds when $d_{X\times Y}$ is the *p*-product metric for 1≤p≤∞.

The objective $\Omega_{k,\rho }\left(\mu_{m},\nu_{n}\right)$ is estimated by its entropy regularized approximation $\Omega_{k,\zeta ,\rho }\left(\mu_{m},\nu_{n}\right)$ for efficient computation, where ζ is the regularization parameter, as follows:

(3) $$\begin{array}{c} \Omega_{k,\zeta ,\rho }\left(\mu_{m},\nu_{n}\right)=W_{k,\zeta }^{k}\left(\mu ,\mu_{m}\right)+W_{k,\zeta }^{k}\left(\nu ,\nu_{n}\right)+\rho W_{k,\zeta }^{k}\left(\gamma_{\lambda }\left(\mu ,\nu \right),\gamma_{\lambda }\left(\mu_{m},\nu_{n}\right)\right). \end{array}$$

Here, $W_{k}^{k}\left(\mu ,\mu_{m}\right)={\langle d_{X}^{k},\gamma \left(\mu ,\mu_{m}\right)\rangle }^{1/k}$ is estimated by $W_{k,\zeta }^{k}\left(\mu ,\mu_{m}\right)={\langle d_{X}^{k},\gamma_{\zeta }\left(\mu ,\mu_{m}\right)\rangle }^{1/k}$. $\gamma_{\zeta }\left(\mu ,\mu_{m}\right)$ is computed by optimizing ${\hat{W}}_{k,\zeta }^{k}\left(\mu ,\mu_{m}\right)\;=\;\langle d_{X}^{k},\gamma_{\zeta }\left(\mu ,\mu_{m}\right)\rangle +\lambda \mathrm{KL}(\gamma_{\zeta }\left(\mu ,\mu_{m}\right)||\mu ⊗\mu_{m}).$

One major difficulty in optimizing $\Omega_{k,\zeta ,\rho }\left(\mu_{m},\nu_{n}\right)$ is to evaluate $W_{k,\zeta }^{k}\left(\gamma_{\lambda }\left(\mu ,\nu \right),\gamma_{\lambda }\left(\mu_{m},\nu_{n}\right)\right)$. In fact, obtaining $\gamma_{\lambda }\left(\mu ,\nu \right)$ is intractable, which is the original motivation for the discretization. To overcome this drawback, by utilizing the dual formulation of EOT, the following are shown (see proof in Appendix A):

**Proposition** **1.** *When X and Y are two compact spaces, and the cost function c is $C^{\infty }$, there exists a constant $C_{1}\in R^{+}$ such that*

$$\begin{array}{c} \max\left\{W_{k}^{k}\left(\mu ,\mu_{m}\right),\;W_{k}^{k}\left(\nu ,\nu_{n}\right)\right\}\;\leq \;W_{k,\zeta }^{k}\;\left(\gamma_{\lambda }\left(\mu ,\nu \right),\;\gamma_{\lambda }\left(\mu_{m},\nu_{n}\right)\right)\leq C_{1}\left[W_{k,\zeta }^{k}\left(\mu ,\mu_{m}\right)+W_{k,\zeta }^{k}\left(\nu ,\nu_{n}\right)\right]. \end{array}$$

Notice that Proposition 1 indicates that $W_{k,\zeta }^{k}\;\left(\gamma_{\lambda }\left(\mu ,\nu \right),\;\gamma_{\lambda }\left(\mu_{m},\nu_{n}\right)\right)$ is bounded above by multiples of $W_{k,\zeta }^{k}\left(\mu ,\mu_{m}\right)+W_{k,\zeta }^{k}\left(\nu ,\nu_{n}\right)$, i.e., when the continuous marginals μ and ν are properly approximated, so is the optimal transference plan between them. Therefore, to optimize $\Omega_{k,\zeta ,\rho }\left(\mu_{m},\nu_{n}\right)$, we focus on developing algorithms to obtain $\mu_{m}^{*},\nu_{n}^{*}$ that minimize $W_{k,\zeta }^{k}\left(\mu ,\mu_{m}\right)$ and $W_{k,\zeta }^{k}\left(\nu ,\nu_{n}\right)$.

**Remark** **1.** *The regularizing parameters (λ and ζ above) introduce smoothness, together with an error term, into the OT problem. To make an accurate approximation, we need λ and ζ to be as small as possible. However, when parameters become too small, the matrices to be normalized in the Sinkhorn algorithm lead to an overflow or underflow problem of numerical data types (32-bit or 64-bit floating point numbers). Thus, the value for regularizing the constant threshold is proportional to the k-th power of the diameter of the supported region. In this work, we try our best to control the value (mainly on ζ), which ranges from 10^−4^ to 0.01 when the diameter is 1 in different examples.*

## 3. Gradient of the Objective Function

Let $\nu =\sum_{i=1}^{m}w_{i}\delta_{y_{i}}$ be a discrete probability measure in the position of “$\mu_{m}$” in the last section. For a fixed (continuous) μ, the objective now is to obtain a discrete target $\nu^{*}=\mathrm{argmin}\;W_{k,\zeta }^{k}\left(\mu ,\nu \right)$.

In order to apply a stochastic gradient descent (SGD) to both the positions ${\left\{y_{i}\right\}}_{i=1}^{m}$ and their weights ${\left\{w_{i}\right\}}_{i=1}^{m}$ to achieve $\nu^{*}$, we now derive the gradient of $W_{k,\zeta }^{k}\left(\mu ,\nu \right)$ about ν by following the discrete discussions of [13,14]. The SGD on *X* is either derived through an exponential map, or by treating *X* as (part of) an Euclidean space.

Let $g\left(x,y\right):=d_{X}^{k}\left(x,y\right)$, and denote the joint distribution minimizing ${\hat{W}}_{k,\zeta }^{k}$ as π with the differential form at $\left(x,y_{i}\right)$ being $d\pi_{i}\left(x\right)$, which is used to define $W_{k,\zeta }^{k}$ in Section 2.

By introducing the Lagrange multipliers $\alpha \in L^{\infty }\left(X\right),\beta \in R^{m}$i, we have ${\hat{W}}_{k,\zeta }^{k}\left(\mu ,\nu \right)=\max_{\alpha ,\beta }$L(μ,ν;α,β), where $L\left(\mu ,\nu ;\alpha ,\beta \right)=\int_{X}\alpha \left(x\right)d\mu \left(x\right)+\sum_{i=1}^{n}\beta w_{i}-\zeta \int_{X}\sum_{i=1}^{n}w_{i}E_{i}\left(x\right)d\mu \left(x\right)$ with $E_{i}\left(x\right)=e^{(\alpha \left(x\right)+\beta_{i}g\left(x,y_{i}\right))/\zeta }$ (see [5]). Let $\alpha^{*},\beta^{*}$ be the argmax; then, we have

$$W_{k,\zeta }^{k}\left(\mu .\nu \right)=\int_{X}\sum_{i=1}^{n}g\left(x,y_{i}\right)E_{i}^{*}\left(x\right)w_{i}d\mu \left(x\right)$$

with $E_{i}^{*}\left(x\right)=e^{(\alpha^{*}\left(x\right)+\beta_{i}^{*}-g\left(x,y_{i}\right))/\zeta }$. Since $\alpha^{\prime }\left(x\right):=\alpha \left(x\right)+t$ and $\beta_{i}^{\prime }:=\beta_{i}-t$ produce the same $E_{i}\left(x\right)$ for any t∈R, the representative with $\beta_{n}=0$ that is equivalent to β (as well as $\beta^{*}$) is denoted by $\bar{\beta }$ (similarly ${\bar{\beta }}^{*}$) below in order to obtain uniqueness and make the differentiation possible.

From a direct differentiation of $W_{k,\zeta }^{k}$, we have

(4) $$\begin{array}{l} \frac{\partial W_{k,\zeta }^{k}}{\partial w_{i}}=\int_{X}g\left(x,y_{i}\right)E_{i}^{*}\left(x\right)d\mu \left(x\right)+\\ \frac{1}{\zeta }\int_{X}\sum_{j=1}^{n}g\left(x,y_{j}\right)\left(\frac{\partial \alpha^{*}\;\left(x\right)}{\partial w_{i}}+\frac{\partial \beta_{j}^{*}}{\partial w_{i}}\right)w_{j}E_{j}^{*}\left(x\right)d\mu \left(x\right). \end{array}$$

(5) $$\begin{array}{c} \nabla_{y_{i}}\;W_{k,\zeta }^{k}=\;\int_{X}\;\;\;\nabla_{y_{i}}g\left(x,y_{i}\right)\left(\;1-\frac{g(x,y_{i})}{\zeta }\;\right)\;E_{i}^{*}\left(x\right)w_{i}d\mu \left(x\right)+\\ \frac{1}{\zeta }\;\;\int_{X}\sum_{j=1}^{n}g\left(x,y_{j}\right)\;\left(\nabla_{y_{i}}\;\alpha^{*}\;\left(x\right)\;+\;\nabla_{y_{i}}\;\beta_{j}^{*}\right)w_{j}E_{j}^{*}\left(x\right)d\mu \left(x\right). \end{array}$$

With the transference plan $d\pi_{i}\left(x\right)=w_{i}E_{i}^{*}\left(x\right)d\mu \left(x\right)$ and the derivatives of $\alpha^{*}$, $\beta^{*}$, $g(x,y_{i})$ calculated, the gradient of $W_{k,\zeta }^{k}$ can be assembled.

Assume that *g* is a Lipschitz constant that is differentiable almost everywhere (for k≥1 and a $d_{X}$ Euclidean distance in $R^{d}$, differentiability fails to hold only when k=1 and $y_{i}=x$) and that $\nabla_{y}g\left(x,y\right)$ is calculated. The derivatives of $\alpha^{*}$ and ${\bar{\beta }}^{*}$ can then be calculated thanks to the Implicit Function Theorem for Banach spaces (see [15]).

The maximality of L at $\alpha^{*}$ and ${\bar{\beta }}^{*}$ induces $N:=\nabla_{\alpha ,\bar{\beta }}{L|}_{\left(\alpha^{*},{\bar{\beta }}^{*}\right)}=0\in {\left(L^{\infty }\left(X\right)⊗R^{m-1}\right)}^{\vee }$, and the Fréchet derivative vanishes. By differentiating (in the sense of Fréchet) again on $\left(\alpha ,\bar{\beta }\right)$ and $y_{i},w_{i}$, respectively, we get

(6) $$\nabla_{\left(\alpha ,\bar{\beta }\right)}N=-\frac{1}{\zeta }\left[\begin{array}{cc} d\mu \left(x\right)\delta \left(x,x^{\prime }\right) & d\pi_{j}\left(x^{\prime }\right)\\ d\pi_{i}\left(x\right) & w_{i}\delta_{ij} \end{array}\right]$$

as a bilinear functional on $L^{\infty }\left(X\right)\times R^{m-1}$ (note that, in Equation (6), the index *i* of $d\pi_{i}$ cannot be *m*). The bilinear functional $\nabla_{\left(\alpha ,\bar{\beta }\right)}N$ is invertible, and we denote its inverse by M as a bilinear form on ${\left(L^{\infty }\left(X\right)⊗R^{m-1}\right)}^{\vee }$. The last ingredient for the Implicit Function Theorem is $\nabla_{w_{i},y_{i}}N$:

(7) $$\nabla_{w_{i}}N=\left(-\frac{1}{w_{i}}\int_{X}\left(\;\cdot \;\right)d\pi_{i}\left(x\right),\vec{0}\right)$$

(8) $$\begin{array}{c} \nabla_{y_{i}}N=\left(\frac{1}{\zeta }\int_{X}\left(\cdot \right)\nabla_{y_{i}}g\left(x,y_{i}\right)d\pi_{i}\left(x\right),\right.\left.\frac{\delta_{ij}}{\zeta }\int_{X}\nabla_{y_{i}}g\left(x,y_{i}\right)d\pi_{i}\left(x\right)\right). \end{array}$$

Then, $\nabla_{w_{i},y_{i}}\left(\alpha^{*},{\bar{\beta }}^{*}\right)=M\left(\nabla_{w_{i},y_{i}}N\right)$. Therefore, we have gradient $\nabla_{w_{i},y_{i}}W_{k,\zeta }^{k}$ calculated.

Moreover, we can differentiate Equations (4)–(8) to get a Hessian matrix of $W_{k,\zeta }^{k}$ on $w_{i}$’s and $y_{i}$’s to provide a better differentiability of g(x,y) (which may enable Newton’s method, or a mixture of Newton’s method and minibatch SGD to accelerate the convergence). More details about the claims, calculations, and proofs are provided in the Appendix B.

## 4. The Discretization Algorithm

Here, we provide a description of an algorithm for the efficient discretizations of optimal transport (EDOT) from a distribution μ to $\mu_{m}$ with integer *m*, which is a given cardinality of support. In general, μ does not need not be explicitly accessible, and, even if it is accessible, computing the exact transference plan is not feasible. Therefore, in this construction, we assume that μ is given in terms of a random sampler, and we apply a minibatch stochastic gradient descent (SGD) through a set of samples that are independently drawn from μ of size *N* on each step to approximate μ.

To calculate the gradient $\nabla_{\mu_{m}}W_{k,\zeta }^{k}\left(\mu ,\mu_{m}\right)={\left(\nabla_{x_{i}}W_{k,\zeta }^{k}\left(\mu ,\mu_{m}\right),\nabla_{w_{i}}W_{k,\zeta }^{k}\left(\mu ,\mu_{m}\right)\right)}_{i=1}^{m}$, we need: (1).  πX,ζ|, the EOT transference plan between μ and $\mu_{m}$, (2).  the cost g=dX|k on *X*, and (3).  its gradient on the second variable ∇x′dX|k(x,x′). From *N* samples ${\left\{y_{i}\right\}}_{i=1}^{N}$, we can construct μN|=1N∑i=1Nδyi and calculate the gradients with μ replaced by μN| as an estimation, whose effectiveness (convergence as N→∞) is proved in [5].

We call this discretization algorithm the *Simple EDOT* algorithm. The pseudocode is stated in the Appendix C.

**Proposition** **2.** (Convergence of the Simple EDOT). *The Simple EDOT generates a sequence $\left(\mu_{m}^{\left(i\right)}\right)$ in the compact set $X^{m}\times \Delta$. If the set of limit points of $\left(\mu_{m}^{\left(i\right)}\right)$ does not intersect with $X^{m}\times \partial \Delta$, then $\left(\mu_{m}^{\left(i\right)}\right)$ converges to a stationary point in $X^{m}\times Int\left(\Delta \right)$ where Int(·) represents the interior.*

In simulations, we fixed k=2 to reduce the computational complexity and fixed the regularizer ζ=0.01 for *X* of diameter 1 and scales proportional with $\mathrm{diam}{\left(X\right)}^{k}$ (see next section). Such a choice for ζ is not only small enough to reduce the error between the EOT estimation $W_{k,\zeta }$ and the true $W_{k}$, but also ensures that $e^{-g(x,y)/\zeta }$ and its byproduct in the SK are distinguishable from 0 in a *double* format.

**Examples of discretization:** We demonstrated our algorithm on the following:

E.g., (1). μ is the uniform distribution on X=[0,1].

E.g., (2) μ is the mixture of two truncated normal distributions on X=[0,1], and the PDF is f(x)=0.3ϕ(x;0.2,0.1)+0.7ϕ(x;0.7,0.2), where ϕ(x;ξ,σ) is the density of the truncated normal distribution on [0,1] with the expectation ξ and standard deviation σ.

E.g., (3) μ is the mixture of two truncated normal distributions on $X={\left[0,1\right]}^{2}$, where the two distributions are ϕ(x;0.2,0.1)ϕ(y;0.3,0.2) of weight 0.3 and ϕ(x;0.7,0.2)ϕ(y;0.6,0.15) of weight 0.7.

**Figure 2:**
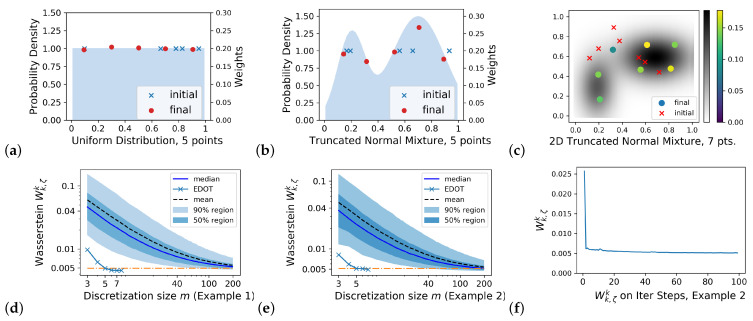
(**a**–**c**) Plots of EDOT discretizations of the Examples (1)–(3). In (**c**), the *x*-axis and *y*-axis are the 2D coordinates, and the probability density of μ and weights of $\mu_{m}$ are encoded by color. (**d**,**e**) show comparison between EDOT and i.i.d. sampling for Examples (1) and (2). EDOT are calculated with m=3 to 7 (3 to 8). The 4 boundary curves of the shaded region are 5%-, 25%-, 75%-, and 95%-percentile curves; the orange line represents the level of m=5; (**f**) plots the entropy regularized Wasserstein distance $W_{k,\zeta }^{k}\left(\mu ,\mu_{m}\right)$ versus the SGD steps for Example (2) with $\mu_{m}$ optimized by 5-point EDOT. ζ=0.01 in all cases.

Let N=100 for all plots in this section. Figure 2a–c plots the discretizations ($\mu_{m}$) for E.g., (1)–(3) with m=5,5, and 7, respectively.

Figure 2f illustrates the convergence rate of $W_{k,\zeta }^{k}\left(\mu ,\mu_{m}\right)$ versus the SGD steps for Example (2) with $\mu_{m}$ obtained by a 5-point EDOT. Figure 2d,e plot the entropy-regularized Wasserstein $W_{k,\zeta }^{k}\left(\mu ,\mu_{m}\right)$ versus *m*, thereby comparing EDOT and naive sampling for Examples (1) and (2). Here, the $\mu_{m}$s are: (a) from the EDOT with 3≤m≤7 in Example 1 and 3≤m≤8 in Example 2, which are shown by ×s in the figures. (b) from naive sampling, which is simulated using a Monte Carlo of volume 20,000 on each size from 3 to 200. Figure 2d,e demonstrate the effectiveness of the EDOT: as indicated by the orange horizontal dashed line, even 5-point EDOT discretization in these two examples outperformed 95% of the naive samplings of size 40, as well as 75% of the naive samplings of size over 100 (the orange dash and dot lines).

**Figure 3:**
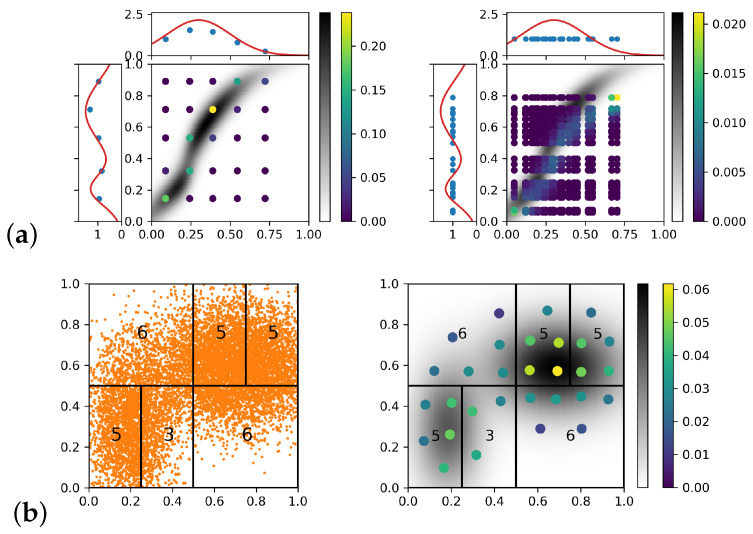
(**a**): Approximation of a transference plan. **Left**: 5×5 EDOT approximation. **Right**: 25×25 naive approximation. In both figures, magnitudes of each point is color coded, the background grayscale density represents the true EOT plan. (**b**): An example of adaptive refinement on a unit square. Left: division of 10,000 sample *S* approximating a mixture of two truncated Gaussian distributions and the refinement for 30 discretization points. Number of discretization points assigned to each region is marked by black numbers. E.g., upper left regaion needs 6 points. Right: the discretization optimized locally and combined as a probability measure with k=2.

**An example of a transference plan:** In Figure 3a, we illustrate the efficiency of the EDOT on an OT problem: X=Y=[0,1], where the marginal μ and ν are truncated normal (mixtures), and μ has two components (shown in red curve on the left), while ν has only one component (shown in red curve on the top). The cost function is the squared Euclidean distance, and λ=ζ=0.01.

The left of Figure 3a shows a 5×5 EDOT approximation with $W_{k,\zeta }^{k}\left(\mu ,\mu_{5}\right)=4.792\times 10^{-3}$, $W_{k,\zeta }^{k}\left(\nu ,\nu_{5}\right)$$=5.034\times 10^{-3}$, and $W_{k,\zeta }^{k}\left(\gamma ,\gamma_{5,5}\right)=8.446\times 10^{-3}$. The high density area of the EOT plan is correctly covered by EDOT estimating points with high weights. The right shows a 25×25 naive approximation with $W_{k,\zeta }^{k}\left(\mu ,\mu_{7}\right)=5.089\times 10^{-3}$, $W_{k,\zeta }^{k}\left(\nu ,\nu_{7}\right)=2.222\times 10^{-2}$, and $W_{k,\zeta }^{k}\left(\gamma ,\gamma_{7,7}\right)=2.563\times 10^{-2}$. The points of the naive estimating with the highest weights missed the region where the true EOT plan was of the most density.

## 5. Methods of Improvement

I. Adaptive EDOT: The computational cost of a simple EDOT increases with the dimensionality and diameter of the underlying space. Discretization with a large *m* is needed to capture higher dimensional distributions, which result an increase in parameters for calculating the gradient of $W_{k,\zeta }^{k}$: md for the $y_{i}$ positions and m−1 for the $w_{i}$ weights. Such an increment will not only increase the complexity in each step, but also require more steps for the SGD to converge. Furthermore, the calculation will have a higher complexity (O(mN) for each normalization in Sinkhorn).

We proposed to reduce the computational complexity using a “divide and conquer” approach. The Wasserstein distance took the *k*-th power of the distance function dX|k as a cost function. The locality of distance dX| made the solution to the OT/EOT problem local, meaning that the probability mass was more likely to be transported to a close destination than to a remote one. Thus, we can “divide and conquer”—thereby cutting the space *X* into small cells and solve the discretization problem independently.

To develop a “divide and conquer” algorithm, we need: (1) an adaptive dividing procedure that is able to partition $X=X_{1}⊔\cdots ⊔X_{I}$, which balances the accuracy and computational intensity among the cells; (2) to determine the discretization size $m_{i}$ and choose a proper regularizer $\zeta_{i}$ for each cell $X_{i}$. The pseudocodes for all variations are shown in the Appendix C Algorithms A2 and A3.

Choosing size *m*: An appropriate choice of $m_{i}$ will balance contributions to the Wasserstein among the subproblems as follows: Let $X_{i}$ be a manifold of dimension *d*, let $\mathrm{diam}(X_{i})$ be its diameter, and let $p_{i}=\mu \left(X_{i}\right)$ be the probability of $X_{i}$. The entropy-regularized Wasserstein distance can be estimated as $W_{k,\zeta }^{k}=O\left(p_{i}m_{i}^{-k/d}\mathrm{diam}{\left(X_{i}\right)}^{k}\right)$ [16,17]. The contribution to $W_{k,\zeta }^{k}\left(\mu ,\mu_{m}\right)$ per point in support of $\mu_{m}$ is $O(p_{i}m_{i}^{-(k+d)/d}\mathrm{diam}{\left(X_{i}\right)}^{k})$. Therefore, to balance each point’s contribution to the Wasserstein among the divided subproblems, we set $m_{i}\approx \frac{{\left(p_{i}\mathrm{diam}{\left(X_{i}\right)}^{k}\right)}^{d/(k+d)}}{\sum_{j=1}^{I}{\left(p_{j}\mathrm{diam}{\left(X_{j}\right)}^{k}\right)}^{d/(k+d)}}$.

Occupied volume (Variation 1):A cell could be too vast (e.g., large in size with few points in a corner), thus resulting in obtaining a larger $m_{i}$ than needed. To fix it, we may replace the $\mathrm{diam}(X_{i})$ above with $\mathrm{Vol}{\left(X_{i}\right)}^{1/d}$, where $\mathrm{Vol}(X_{i})$ is the occupied volume calculated by counting the number of nonempty cells in a certain resolution (levels in previous binary division). The algorithm (Variation 1) becomes a binary tree to resolve and obtain the occupied volume for each cell, then there is tree traversal to assign $m_{i}$.

Adjusting the regularizer ζ: In the $W_{k,\zeta }^{k}$, the SK on $e^{-g(x,y)/\zeta }$ is calculated. Therefore, ζ should scale with $d_{X}^{k}$ to ensure that the transference plan is not affected by the scaling of $d_{X}$. Precisely, we may choose $\zeta_{i}=\mathrm{diam}{\left(X_{i}\right)}^{k}\zeta_{0}$ for some constant $\zeta_{0}$.

The division: Theoretically, any refinement procedure that proceeds iteratively and eventually makes the diameter of each cell approach 0 can be applied for division. In our simulation, we used an adaptive kd-tree-style cell refinement in a Euclidean space $R^{d}$. Let *X* be embedded into $R^{d}$ within an axis-aligned rectangular region. We chose an axis $x_{l}$ in $R^{d}$ and evenly split the region along a hyperplane orthogonal to $x_{l}$ (e.g., cut square ${\left[0,1\right]}^{2}$ along the line x=0.5); thus, we constructed $X_{1}$ and $X_{2}$. With the sample set *S* given, we split it into two sample sizes $S_{1}$ and $S_{2}$ according to which subregion each sample was located in. Then, the corresponding $m_{i}$ and $\zeta_{i}$ could be calculated as discussed above. Thus, two cells and their corresponding subproblems were constructed. If some of the $m_{i}$ was still too large, the cell was cut along another axis to construct two other cells. The full list of cells and subproblems could be constructed recursively. In addition, another cutting method (variation 2) that chooses the most sparse point as a cutting point through a sliding window is sometimes useful in practice.

After having the set of subproblems, we could apply the EDOT for the solutions in each cell, then combine the solutions μmi(i)=∑j=1miwj(i)δyj(i)| into the final result μm:=∑i=1I∑j=1mipiwj(i)δyj(i)|.

Figure 3b shows the optimal discretization for the example in Figure 2c with m=30, which was obtained by applying the EDOT with adaptive cell refinement, or $\zeta =0.01\times {\mathrm{diam}}^{2}$.

II. On embedded CW complexes: Although the samples on space *X* are usually represented as a vector in $R^{d}$, inducing an embedding $X↪R^{d}$, the space *X* usually has its own structure as a CW complex (or simply a manifold) with a more intrinsic metric. Thus, if the CW complex structure is known, even piecewise, we may apply the refinement on *X* with respect to its own metric, whereas direct discretization as a subset in $R^{d}$ may result in a low expressing efficiency.

We now illustrate the adaptive EDOT by an example on a mixture normal distribution of a sphere mapped through stereographic projection. More examples of a truncated normal mixture over a Swiss roll and the discretization of a 2D optimal transference plan are detailed in the Appendix D.5.

**Figure 4:**
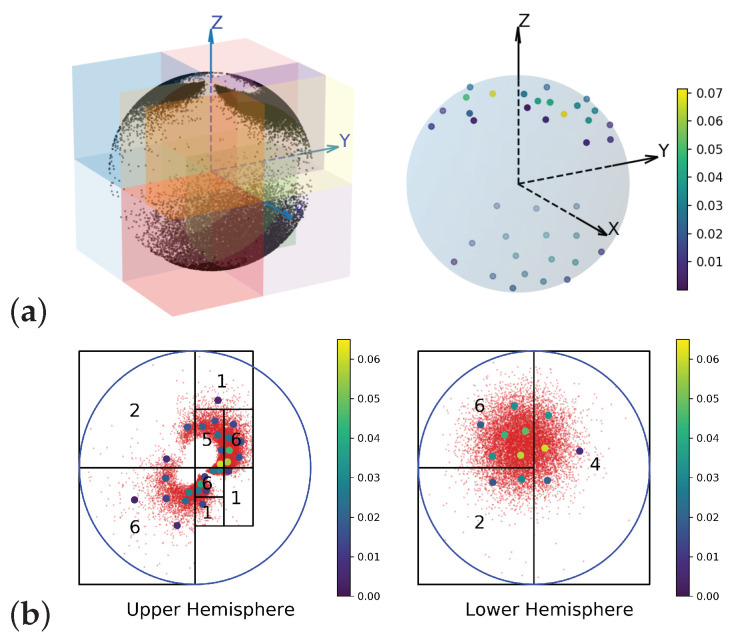
(**a**) **Left**: 30,000 samples from $\mu_{sphere}$ and the 3D cells under divide-and-conquer algorithm **Right**: 40-point EDOTs in 3D. (**b**) The 40-point CW-EDOTs in 2D. Red dots: samples, other dots: discrete atoms with weights represented in colors. **Left**: upper hemisphere. **Right**: lower hemisphere, stereographic projections about poles. $\zeta =0.01\times {\mathrm{diam}}^{2}$.

On the sphere: The underlying space $X_{\mathrm{sphere}}$ is the unit sphere in $R^{3}$. $\mu_{\mathrm{sphere}}$ is the pushforward of a normal mixture distribution on $R^{2}$ by stereographic projection. The sample set $S_{\mathrm{sphere}}∼\mu_{\mathrm{sphere}}$ over $X_{\mathrm{sphere}}$ is shown on Figure 4 on the left. Consider a (3D) Euclidean metric on the $X_{\mathrm{sphere}}$ induced by the embedding. Figure 4a (right) plots the EDOT solution with refinement for $\mu_{m}$ with m=40. The resulting cell structure is shown as colored boxes.

To consider the intrinsic metric, a CW complex was constructed about a point on the equator as a 0-cell structure; the rest of the equator was constructed as a 1-cell, and the upper hemisphere and lower hemisphere were constructed as two dimension 2- (open) cells. We took the upper and lower hemispheres and mapped them onto a unit disk through stereographic projection with respect to the south and north pole, respectively. Then, we took the metric from spherical geometry and rewrote the distance function and its gradient using the natural coordinate on the unit disk. Figure 4b shows the refinement of the EDOT on the samples (in red) and the corresponding discretizations in colored points. More figures can be found in the Appendices.

## 6. Analysis of the Algorithms

In this section, we derive the complexity of the simple EDOT and the adaptive EDOT. In particular, we show the following:

**Proposition** **3.** *Let μ be a (continuous) probability measure on a space X. A simple EDOT of size m has time complexity $O({\left(N+m\right)}^{2}mdL+NmL\log\left(1/\epsilon \right))$ and space complexity $O({\left(N+m\right)}^{2})$, where N is the minibatch size (to construct $\mu_{N}$ in each step to approximate μ), d is the dimension of X, L is the maximal number of iterations for SGD, and ϵ is the error bound in the Sinkhorn calculation for the entropy-regularized optimal transference plan between $\mu_{N}$ and $\mu_{m}$.*

Proposition 3 quantitatively shows that, when the adaptive EDOT is applied, the total complexities (in time and space) are reduced, because the magnitudes of both *N* and *m* are much smaller in each cell.

The procedure of dividing sample set *S* into subsets through the adaptive EDOT is similar to Quicksort; thus, the space and time complexities are similar. The similarity comes from the binary divide-and-conquer structure, as well as that each split action is based on comparing each sample with a target.

**Proposition** **4.** *For the preprocessing (job list creation) for the adaptive EDOT, the time complexity is $O(N_{0}\log N_{0})$ in the best and average case and $O(N_{0}^{2})$ in the worst case, where $N_{0}$ is the total number of sample points, and the space complexity is $O(N_{0}d+m)$, or simply $O(N_{0}d)$ as $m\ll N_{0}$.*

**Remark** **2.** *Complexity is the same as Quicksort. The set of $N_{0}$ sample points in the algorithm are treated as the “true” distribution in the adaptive EDOT, since, in the later EDOT steps for each cell, no further samples are taken, as it is hard for a sampler to produce a sample in a given cell. Postprocessing of the adaptive EDOT has O(m) complexity in both time and space.*

**Remark** **3.** *For the two algorithm variations in Section 5, the occupied volume estimation works in the same way as the original preprocessing step, which has the same time complexity as before (by itself, since dividing must happen after knowing the occupied volume of all cells), but, with the tree built, the original preporcessing becomes a tree traversal and has (additional) time complexity $O(N_{0})$ and (additional) space complexity $O(N_{0})$ for the space storing occupied volume.*

*For details on choosing cut points with window sliding, the discussion can be seen in the Appendix C.5.*

**Comparison with naive sampling:** After having a size *m* discretization on *X* and a size *n* discretization on *Y*, the EOT solution (Sinkhorn algorithm) has time complexity O(mnlog(1/ϵ)). In the EDOT, two discretization problems must be solved before applying the Sinkhorn, while the naive sampling requires nothing but sampling.

According to Proposition 3, solving a single continuous EOT problem using a size *m* simple EDOT method may result in higher time complexity than naive sampling with an even larger sample size *N* (than *m*). However, unlike the EDOT, which only requires access to a distance function $d_{X}$ and $d_{Y}$ on *X* and *Y*, respectively, a known cost function c:X×Y→R is necessary for naive sampling. In real applications, the cost function may be from real world experiments (or from extra computations) done for each pair (x,y) in the discretization; thus, the size of discretized distribution is critical for cost control. $d_{X}$ and $d_{Y}$ usually come along with the spaces *X* and *Y*, respectively, and are easy to compute. An additional application of the EDOT is necessary when the marginal distributions $\mu_{X}$ and $\nu_{Y}$ are fixed for different cost functions; then, discretizations can be reused. Thus, the cost of discretization is calculated one time, and the improvement it brings accumulates in each repeat.

## 7. Related Work and Discussion

Our original problem was the optimal transport problem between general distributions as samplers (instead of integration oracles). We translated that into a discretization problem and an OT problem between discretizations.

I. Comparison with other discretization methods: There are several other methods that generate discrete distributions from arbitrary distributions in the literature, which are obtained via semi-continuous optimal transport where the calculation of a weighted Voronoi diagram is needed. Calculating the weighted Voronoi diagrams usually requires 1. that the cost function be a squared Euclidean distance and 2. the application of Delaunay triangulation, which is expensive in more than two dimensions. Furthermore, semi-continuous discretization may only optimize one aspect between the position and weights of the atoms, and this process is mainly based on [18] (the optimized position) and [19] (the optimized weights).

**Figure 5:**
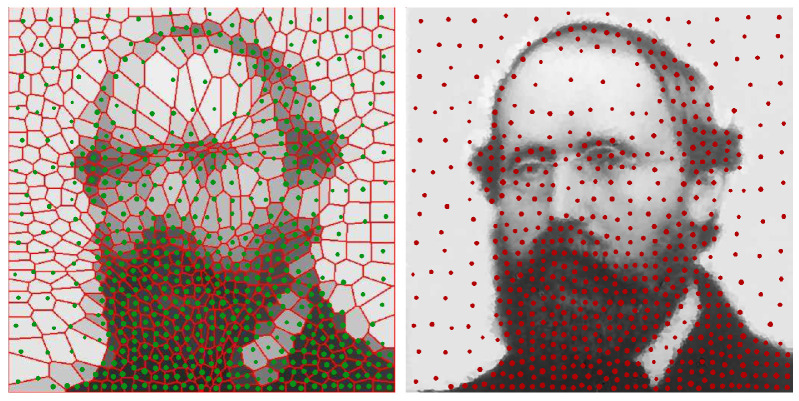
EDOT of an example from [19]. Potrait of Riemann, discretization of size 625. **Left**: green dots show position and weights of EDOT discretization (same as right); cells in background are discretization of the same size in the original [19]. **Right**: A size 10,000 discretization from [19]; we directly applied EDOT to this picture, treating it as the continuous distribution. $\zeta =0.01\times {\mathrm{diam}}^{2}$.

We mainly compared the prior work of [18], which focuses on the barycenter of a set of distributions under the Wasserstein metric. This work resulted in a discrete distribution called the Lagrangian discretization, which is of the form $\frac{1}{m}\sum_{i=1}^{m}\delta_{x_{i}}$ [2]. Other works, such as [20,21], find barycenters but do not create a discretization. Refs. [19,22] studied the discrete estimation of a 2-Wasserstein distance locating discrete points through a clustering algorithm *k-means++* and a weighted Voronoi diagram refinement, respectively. Then, they assigned weights and made them non-Lagrangian discretizations. Ref. [19] (comparison in Figure 5)  roughly followed a “divide-and-conquer” approach in selecting positions, but the discrete positions were not tuned according to Wasserstein distance directly. Ref. [22] converged as the number of discrete points increased. However, it lacked a criterion (such as the Wasserstein in the EDOT) to show that the choice is not just one among all possible converging algorithms, but, rather, it is a special one.

**Figure 6:**
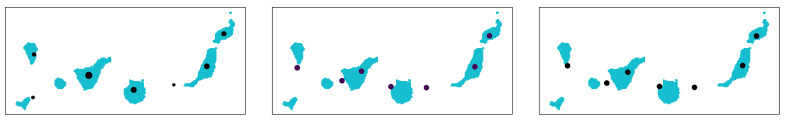
A comparison of EDOT (**left**), EDOT-Equal (**mid**), and [18] (**right**) on the Canary islands, treated as a binary distribution with a constant density on islands and 0 in the sea. Discretizations for each method is shown by black bullets. Wasserstein distances: EDOT: $W_{0.005}^{2}=0.02876$, EDOT-Equal: $W_{0.005}^{2}=0.05210$, Claici: $W_{0.005}^{2}=0.05288$. Map size is 3.13×1.43.

By projecting the gradient in the SGD to the tangent space of the submanifold $X^{m}\times \left\{e_{m}/m\right\}=\left\{\frac{1}{m}\sum \delta_{x_{i}}\right\}$, or by equivalently fixing the learning rate on the weights to zero, the EDOT can estimate a Lagrangian discretization (denoted by EDOT-Equal). A comparison among the methods is held on the map of the Canary islands, which is shown in Figure 6. This example shows that our method can get a similar result using Lagrangian discretization as the methods in the literature, while, in general, this type of EDOT can work better.

Moreover, the EDOT can be used to solve barycenter problems.

Note that, to apply adaptive EDOT for barycenter problems, compatible divisions of the target distributions are needed (i.e., a cell A from one target distribution transports onto a discrete subset *D* thoroughly, and *D* transports onto a cell B from another target distribution, etc.).

**Figure 7:**
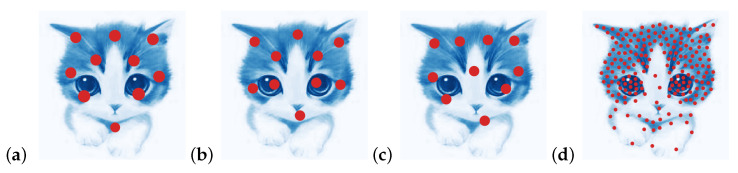
Discretization of a kitty. Discretization by each method is shown in red bullets on top of the Kitty image. (**a**) EDOT, 10 points, $W_{0.001}^{2}=0.009176$, radius represents weight; (**b**) EDOT-Equal, 10 points, $W_{0.001}^{2}=0.008960$; (**c**) [18], 10 points, $W_{0.001}^{2}=0.009832$; (**d**) [18], 200 points. Figure size 1×1, $\zeta =0.01\times {\mathrm{diam}}^{2}$.

**Figure 8:**
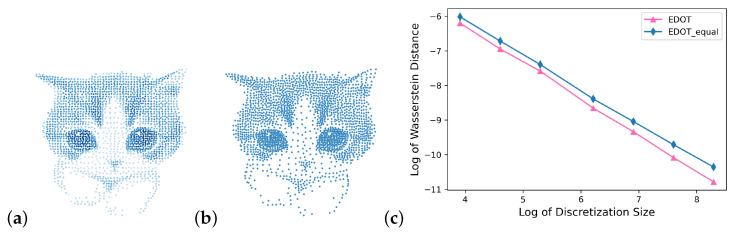
2000-Point Discretizations, (**a**). EDOT (weight plotted in color), (**b**). EDOT-Equal, (**c**). Relations between $\log(W^{2})$ and logm (all with divide and conquer); it can be seen that the advantage of $W_{\mathrm{EDOT}}$ over $W_{\mathrm{Equal}}$ grows with the size of discretization.

We also tested these algorithms on discretizing gray/colored scale pictures. The comparison of discretization with points varying from 10 to 4000 for a kitty image between EDOT, EDOT-equal, [18] and estimations of their Wasserstein distances to the original image are shown in Figure 7 and Figure 8.

Furthermore, the EDOT may be applied on RGB channels of an image independently, which then combine plots of discretizations in the corresponding color. The results are shown in Figure 1 at the beginning of this paper.

Lagrangian discretization may have a disadvantage in representing repetitive patterns with incompatible discretization points.

**Figure 9:**
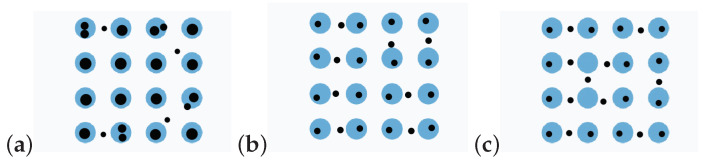
Discretization of 16 blue disks in a unit square with 24 points (black). (**a**) EDOT, $W_{\zeta }^{2}=0.001398$; (**b**) EDOT-Equal, $W_{\zeta }^{2}=0.002008$; (**c**) [18], $W_{\zeta }^{2}=0.002242$. $\zeta =10^{-4}$. Figure size is 1×1.

In Figure 9, we can see that discretizing 16 objects with 24 points caused weight incompatibility locally for the Lagrangian discretization, thus making points locate between objects and increasing the Wasserstein distance. With the EDOT, the weights of points that lie outside of the blue object were much smaller. The patterned structure was better represented by the EDOT. In practice, patterns often occur as part of the data (e.g., pictures of nature), and it is easy to get an incompatible number in Lagrangian discretization, since the equal weight-requirement is rigid; consequently, patterns cannot be properly captured.

II. General *k* and deneral distance $d_{X}$: Our algorithms (Simple EDOT, adaptive EDOT, and EDOT-Equal) work for a general choice of parameter k>1 and $C^{2}$ distance $d_{X}$ on *X*. For example, in Figure 4 part (b), the distance used on each disk was spherical (arc length along the big circle passing through two points), which could not be isometrically reparametrized into a plane with Euclidean metrics because of the difference in curvatures.

III. Other possible impacts: As the OT problem widely exists in many other areas, our algorithm can be applied accordingly, e.g., the location and size of supermarkets or electrical substations in an area, or even air conditioners in the rooms of supercomputers. Our divide-and-conquer methods are suitable for solving these real-world applications.

IV. OT for discrete distributions: Many algorithms have been developed to solve OT problems between two discrete distributions [3]. Linear programming algorithms were first developed, but their applications have been restricted by high computational complexity. Other methods such as [23], with a cost of form c(x,y)=h(x−y) for some *h*, which applies the “back-and-forth” method by hopping between two forms of a Kantorovich dual problem (on the two marginals, respectively) to get a gradient of the total cost over the dual functions, usually solve problems with certain conditions. In our work, we chose to apply an EOT developed by [8] for an estimated OT solution of the discrete problem.

## 8. Conclusions

We developed methods for efficiently approximating OT couplings with fixed size m×n approximations. We provided bounds on the relationship between a discrete approximation and the original continuous problem. We implemented two algorithms and demonstrated their efficacy as compared to naive sampling and analyzed computational complexity. Our approach provides a new approach to efficiently compute OT plans.

## Figures and Tables

**Figure A1:**
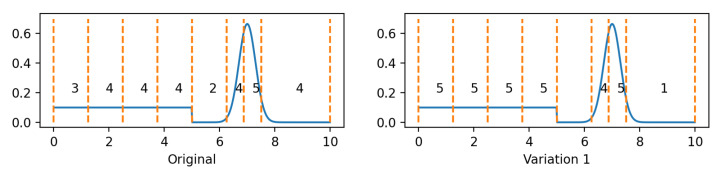
Divide-and-conquer strategies: original and Variation 1. Original tends to assign more atoms to vast region with small weights, while Variation 1 does better. Example: distribution on [0,10], pdf is plotted in blue curve, discretization size of each cell is in black.

**Figure A2:**
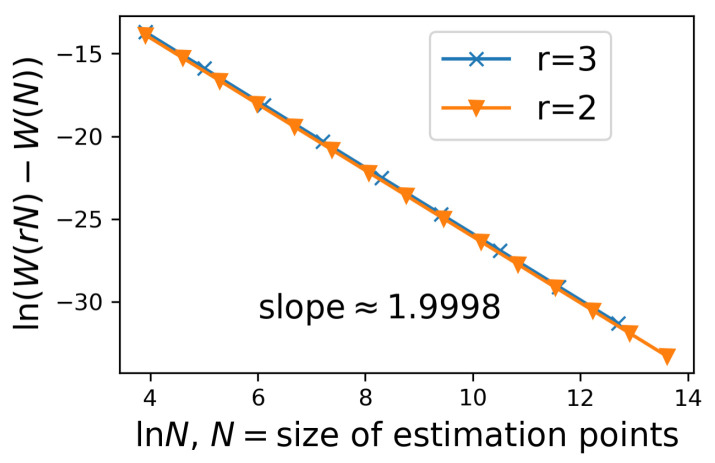
Richardson Extrapolation: the power of the expansion about $N^{-1}$. We take the EDOT $\mu_{5}$ of example 2 (1-dim truncated normal mixture) as the target $\mu_{m}$ and use evenly positioned $\mu_{N}$ for different *N*s to estimate. The *y*-axis is $\ln(W_{2,0.01}^{2}\left(\mu_{\left(rN\right)},\mu_{5}\right)-W_{2,0.01}^{2}\left(\mu_{\left(N\right)},\mu_{5}\right))$, where r=2 and r=3 are calculated. With lnN as *x*-axis, linearity can be observed. The slopes are both about −1.9998, which represent the exponent of the leading non-constant term of $W_{2,0.01}^{2}\left(\mu_{\left(N\right)},\mu_{5}\right)$ on *N*, while the theoretical result is r=−k/d=−2. The differences are from higher order terms on *N*.

**Figure A3:**
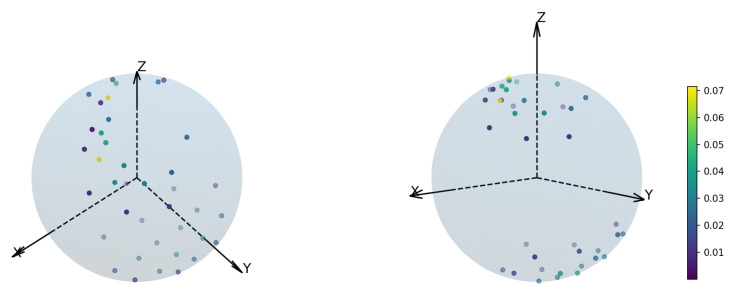
The sphere example with 3D discretization (same as the paper) on two view directions. Colors of dots represent the weights of each atom in the distribution.

**Figure A4:**
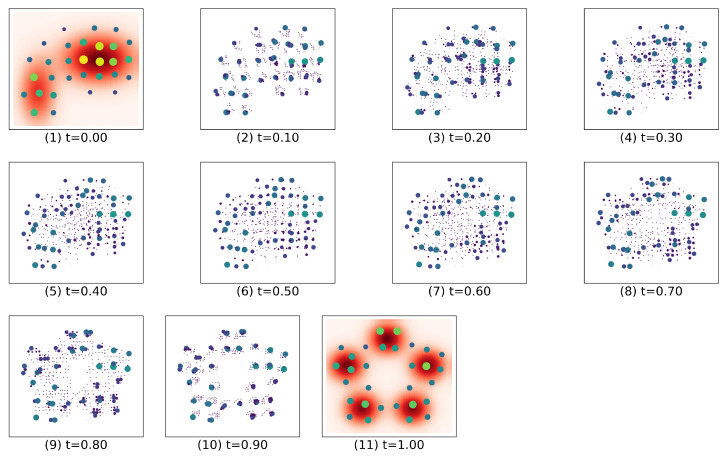
The McCann interpolation figures in finer time resolution for visualizing the transference plan from (**1**)–(**11**). It is a refined figure of the original paper. We see can see that the larger bubbles (representing a large probability mass) moved in a short distance, and smaller pieces moved longer.

**Figure A5:**
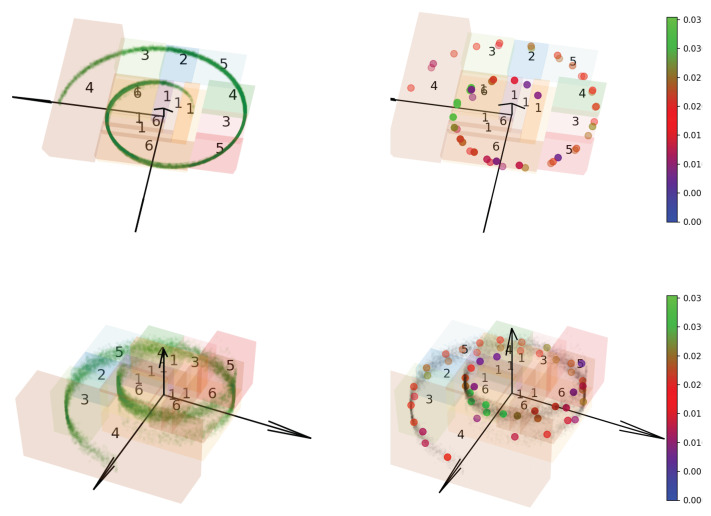
Discretization of a distribution supported on a Swiss Roll. **Left**: A total of 15,000 samples from the truncated normal mixture distribution $\mu_{\mathrm{swiss}}$ over $X_{\mathrm{swiss}}$. **Right**: A 50-point 3D discretization using Variation 2 of Algorithm A2; the refinement cells are shown in colored boxes.

**Figure A6:**
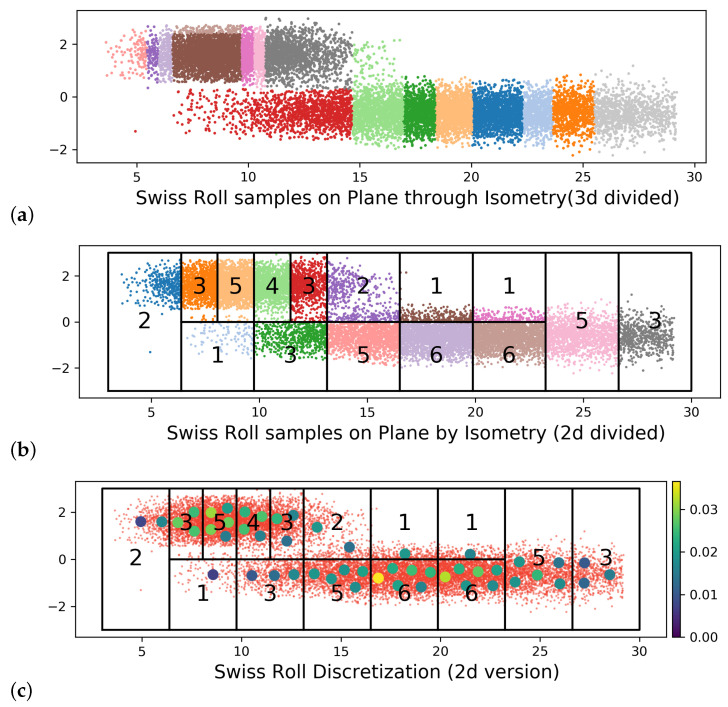
Swiss Roll under isometry. (**a**) Refinement cells under 3D Euclidean metric (one color per samples from a refinement cell). (**b**) Refinement cells under 2D metric induced by the isometry. (**c**) EDOT of 50 points with respect to the 2D refinement. $\zeta =0.01\times {\mathrm{diam}}^{2}$ for all.

**Figure A7:**
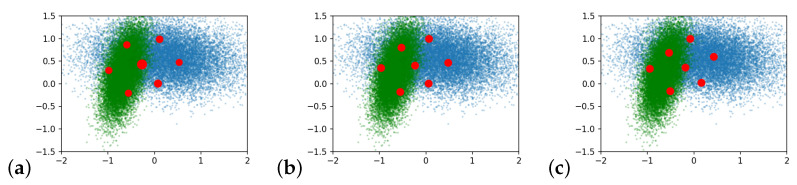
A 7-point barycenter of two Gaussian distributions: (**a**): EDOT, area of dots represent the weights, $W_{sum}^{2}=0.7322$; (**b**): EDOT-Equal, $W_{sum}^{2}=0.7380$; (**c**): [18], $W_{sum}^{2}=0.7389$; *W* are regularized with ζ=0.04.

## Data Availability

Not applicable.

## Abbreviations

The following abbreviations are used in this manuscript:

OT	Optimal Transport
EOT	Entropy-Regularized Optimal Transport
EDOT	Efficient Discretization of Optimal Transport
SGD	Stochastic Gradient Descent

## Appendix A. Proof of Proposition 1

**Proof.** We will adopt the notations Z=X×Y and $z_{i}=\left(x_{i},y_{i}\right)\in Z$. Furthermore, recall the condition:

(A1) $$\begin{array}{c} \max\left\{d_{X}\left(x,x^{\prime }\right),d_{Y}\left(y,y^{\prime }\right)\right\}\leq d_{Z}\left(z,z^{\prime }\right)\leq d_{X}\left(x,x^{\prime }\right)+d_{Y}\left(y,y^{\prime }\right) \end{array}$$

For inequality (i) without loss, assume that

$$\max\left\{W_{k}^{k}\left(\mu ,\mu_{m}\right),W_{k}^{k}\left(\nu ,\nu_{n}\right)\right\}=W_{k}^{k}\left(\mu ,\mu_{m}\right)$$

Denote the optimal $\pi_{Z}\in \Pi \left(\gamma_{\lambda }\left(\mu ,\nu \right),\gamma_{\lambda }\left(\mu_{m},\nu_{n}\right)\right)$ that achieves $W_{k}^{k}\left(\gamma_{\lambda }\left(\mu ,\nu \right),\gamma_{\lambda }\left(\mu_{m},\nu_{n}\right)\right)$ by $\pi_{Z}^{*}$ and similarly for $\pi_{X}^{*}$. Then, we have:

$$\begin{array}{cc} W_{k}^{k} & \left(\gamma_{\lambda }\left(\mu ,\nu \right),\gamma_{\lambda }\left(\mu_{m},\nu_{n}\right)\right)=\int_{Z^{2}}d_{Z}^{k}\left(z_{1},z_{2}\right)d\pi_{Z}^{*}\geq A\int_{Z^{2}}d_{X}^{k}\left(x_{1},x_{2}\right)d\pi_{Z}^{*}\\ \; & =A\int_{X^{2}}\;d_{X}^{k}\left(x_{1},x_{2}\right)\int_{Y^{2}}\;d\pi_{Z}^{*}\overset{\left(a\right)}{=}A\;\int_{X^{2}}\;d_{X}^{k}\left(x_{1},x_{2}\right)d\pi_{X}^{\prime }\\ \; & \overset{\left(b\right)}{\geq }A\int_{X^{2}}d_{X}^{k}\left(x_{1},x_{2}\right)d\pi_{X}^{*}=W_{k}^{k}\left(\mu ,\mu_{m}\right) \end{array}$$

Here, $\pi_{X}^{\prime }\in \Pi \left(\mu ,\mu_{m}\right)$ and eq (a) hold since $\pi_{Z}^{*}\in \Pi \left(\gamma_{\lambda }\left(\mu ,\nu \right),\gamma_{\lambda }\left(\mu_{m},\nu_{n}\right)\right)$, and ineq (b) holds since $\pi_{X}^{*}$ is the optimal choice. For inequality (ii), we use the following to simplify the notations: $d\gamma ⊗d\gamma_{mn}\;:=d\gamma_{\lambda }\left(\mu ,\nu \right)⊗d\gamma_{\lambda }\left(\mu_{m},\nu_{n}\right)$ and $d_{Z}^{k}\;:=d_{Z}^{k}\left(z_{1},z_{2}\right),d_{X}^{k}\;:=d_{X}^{k}\left(x_{1},x_{2}\right),d_{Y}^{k}\;:=d_{Y}^{k}\left(y_{1},y_{2}\right)$

$$\begin{array}{cc} \; & W_{k,\zeta }^{k}\left(\gamma_{\lambda }\left(\mu ,\nu \right),\gamma_{\lambda }\left(\mu_{m},\nu_{n}\right)\right)=\int_{Z^{2}}d_{Z}^{k}\left(z_{1},z_{2}\right)d\pi_{z,\zeta }^{*}\\ \; & \overset{\left(a\right)}{=}\int_{Z^{2}}d_{Z}^{k}\cdot \exp\left(\frac{\alpha \left(z_{1}\right)+\beta \left(z_{2}\right)-d_{Z}^{k}\left(z_{1},z_{2}\right)}{\zeta }\right)d\gamma ⊗d\gamma_{mn}\\ \; & \overset{\left(b\right)}{\leq }A\int_{Z^{2}}\left(d_{X}^{k}+d_{Y}^{k}\right)\cdot \exp\left(\frac{\alpha \left(z_{1}\right)+\beta \left(z_{2}\right)-d_{Z}^{k}}{\zeta }\right)d\gamma ⊗d\gamma_{mn}\\ \; & \overset{\left(c\right)}{\leq }C_{1}\int_{Z^{2}}\left(d_{X}^{k}+d_{Y}^{k}\right)\cdot \exp\left(\frac{-d_{Z}^{k}}{\zeta }\right)d\gamma ⊗d\gamma_{mn}\\ \; & \overset{\left(d\right)}{\leq }C_{1}\left[\int_{Z^{2}}d_{X}^{k}\cdot \exp\left(\frac{-d_{X}^{k}}{\zeta }\right)+d_{Y}^{k}\cdot \exp\left(\frac{-d_{Y}^{k}}{\zeta }\right)d\gamma ⊗d\gamma_{mn}\right]\\ \; & \overset{\left(e\right)}{=}C_{1}\left[\int_{X^{2}}d_{X}^{k}\cdot \exp\left(\frac{-d_{X}^{k}}{\zeta }\right)d\mu ⊗d\mu_{m}+\int_{Y^{2}}d_{Y}^{k}\cdot \exp\left(\frac{-d_{Y}^{k}}{\zeta }\right)d\nu ⊗d\nu_{n}\right]\\ \; & \overset{\left(f\right)}{\leq }C_{1}\int_{X^{2}}d_{X}^{k}\cdot \exp\left(\frac{s\left(x_{1}\right)+t\left(x_{2}\right)-d_{X}^{k}}{\zeta }\right)d\mu ⊗d\mu_{m}\\ \; & \;+C_{1}\int_{Y^{2}}d_{Y}^{k}\cdot \exp\left(\frac{s^{\prime }\left(y_{1}\right)+t^{\prime }\left(y_{2}\right)-d_{Y}^{k}}{\zeta }\right)d\nu ⊗d\nu_{n}\\ \; & =C_{1}\left[W_{k,\zeta }^{k}\left(\mu ,\mu_{m}\right)+W_{k,\zeta }^{k}\left(\nu ,\nu_{n}\right)\right] \end{array}$$

Justifications for the derivations: (a) Based on the dual formulation, it is shown in [5] Proposition 1 that, for ζ>0, there exist $\alpha \left(z_{1}\right),\beta \left(z_{2}\right)\in C\left(Z\right)$ such that: $d\pi_{z,\zeta }^{*}=\exp\left(\frac{\alpha \left(z_{1}\right)+\beta \left(z_{2}\right)-d_{Z}^{k}\left(z_{1},z_{2}\right)}{\zeta }\right)d\gamma ⊗d\gamma_{mn}$; (b) Inequality (ii) of Equation (A1); (c) According to Ref. [24] Theorem 2, when *X* and *Y* are compact and *c* is smooth, α and β are uniformly bounded; moreover, both $d_{X}^{k}$ and $d_{Y}^{k}$ are uniformly bounded by the diameter of *X* and *Y*, respectively; hence, the constant *B* exists; (d) Inequality (ii) of Equation (A1); (e) $\gamma_{\lambda }\left(\mu ,\nu \right)\in \Pi \left(\mu ,\nu \right)$ and $\gamma_{\lambda }\left(\mu_{m},\nu_{n}\right)\in \Pi \left(\mu_{m},\nu_{n}\right)$; (f) Similarly as in (a), for ζ>0, there exist $s\left(x_{1}\right),t\left(x_{2}\right)\in C\left(X\right)$, and $s^{\prime }\left(y_{1}\right),t^{\prime }\left(y_{2}\right)\in C\left(Y\right)$ such that $\exp\left(\frac{-d_{X}^{k}}{\zeta }\right)d\mu ⊗d\mu_{m}=d\pi_{X}^{*}$ and $\exp\left(\frac{-d_{Y}^{k}}{\zeta }\right)d\nu ⊗d\nu_{n}=d\pi_{Y}^{*}$. Moreover, $\int_{X^{2}}d_{X}^{k}\cdot \exp\left(\frac{s\left(x_{1}\right)+t\left(x_{2}\right)}{\zeta }\right)d\mu ⊗d\mu_{m}\geq 0$, and $\int_{Y^{2}}d_{Y}^{k}\cdot \exp\left(\frac{s^{\prime }\left(y_{1}\right)+t^{\prime }\left(y_{2}\right)}{\zeta }\right)d\nu ⊗d\nu_{n}\geq 0$.    □

## Appendix B. Gradient of $W_{k,\zeta }^{k}$

### Appendix B.1. The Gradient

Following the definitions and notations in Section 2 and Section 3 of the paper, we calculate the gradient of $W_{k,\zeta }^{k}\left(\mu ,\mu_{m}\right)$ about parameters of $\mu_{m}:=\sum_{i=1}^{m}w_{i}\delta_{y_{i}}$ in detail.

$W_{k,\zeta }^{k}\left(\mu ,\mu_{m}\right)=\int_{X^{2}}g\left(x,y\right)d\gamma_{\zeta }\left(x,y\right)$, where

(A2) $$\gamma_{\zeta }=\mathrm{argmin}{\;}_{\gamma \in \Pi (\mu ,\mu_{m})}\int_{X^{2}}\;\;\;g\left(x,y\right)d\gamma (x,y)+\zeta \mathrm{KL}(\gamma ||\mu ⊗\mu_{m}).$$

Let $\alpha \in L^{\infty }\left(X\right)$ and $\beta \in R^{m}$. Denote $\beta =\sum_{i=1}^{m}\beta_{i}\delta_{y_{i}}$, and let

(A3) $$\begin{array}{rr} F(\gamma ;\mu ,\mu_{m},\alpha ,\beta ):= & \int_{X^{2}}\;\;\;g\left(x,y\right)d\gamma (x,y)+\zeta \mathrm{KL}(\gamma ||\mu ⊗\mu_{m})\\ \; & +\int_{X}\alpha \left(x\right)\left(\int_{X}d\gamma \left(x,y\right)-d\mu_{m}\left(y\right)\right)\\ \; & +\int_{X}\beta \left(y\right)\left(\int_{X}d\gamma \left(x,y\right)-d\mu \left(x\right)\right)\\ = & \int_{X^{2}}\;\;\;g\left(x,y\right)d\gamma (x,y)+\zeta \mathrm{KL}(\gamma ||\mu ⊗\mu_{m})\\ \; & +\int_{X}\alpha \left(x\right)\left(\sum_{i=1}^{m}d\gamma \left(x,y_{i}\right)-d\mu_{m}\left(y_{i}\right)\right)\\ \; & +\sum_{i=1}^{m}\beta_{i}\left(\int_{X}d\gamma \left(x,y_{i}\right)-d\mu \left(x\right)\right) \end{array}$$

Since γ on the second component *X* is discrete and supported on ${\left\{y_{i}\right\}}_{i=1}^{m}$, we may denote $d\gamma (x,y_{i})$ by $d\pi_{i}\left(x\right)$; thus,

(A4) $$\begin{array}{cc} F(\gamma ;\mu ,\mu_{m},\alpha ,\beta )= & \int_{X}\sum_{i=1}^{m}g\left(x,y_{i}\right)d\pi_{i}\left(x\right)\\ \; & +\zeta \sum_{i=1}^{m}\int_{X}\left(\ln\frac{d\pi_{i}\left(x\right)}{d\mu (x)}-\ln\left(\mu_{m}\left(y_{i}\right)\right)\right)d\pi_{i}\left(x\right)\\ \; & +\int_{X}\alpha \left(x\right)\left(\sum_{i=1}^{m}d\pi_{i}\left(x\right)-d\mu_{m}\left(y_{i}\right)\right)\\ \; & +\sum_{i=1}^{m}\beta_{i}\left(\int_{X}d\pi_{i}\left(x\right)-d\mu \left(x\right)\right) \end{array}$$

Then, the Fenchel duality of Problem (A2) is

(A5) $$\begin{array}{cc} \; & L(\mu ,\mu_{m};\alpha ,\beta )\\ = & \int_{X}\alpha \left(x\right)d\mu \left(x\right)+\sum_{i=1}^{m}\beta_{i}w_{i}-\zeta \int_{X}\sum_{i=1}^{m}e^{(\alpha \left(x\right)+\beta_{i}-g\left(x,y_{i}\right))/\zeta }w_{i}d\mu \left(x\right). \end{array}$$

Let $\alpha^{*}$ and $\beta^{*}$ be the argmax of the Fenchel dual (A5). The primal is solved by $d\gamma^{*}\left(x,y_{i}\right)=e^{(\alpha^{*}\left(x\right)+\beta_{i}^{*}-g\left(x,y_{i}\right))/\zeta }w_{i}d\mu \left(x\right).$ To make the solution unique, we restrict the freedom of the solution (where we see that $L\left(\mu ,\mu_{m};\alpha ,\beta \right)=L\left(\mu ,\mu_{m};\alpha +t,\beta -t\right)$ for any t∈R). We use the condition $\beta_{m}=0$ to narrow the choices down to only one, and denote the dual variable β having the property $\bar{\beta }$ and ${\bar{\beta }}^{*}$.

We first calculate $\nabla_{w_{i},y_{i}}L\left(\mu ,\mu_{m};\alpha^{*},{\bar{\beta }}^{*}\right)$ with $\alpha^{*}$ and ${\bar{\beta }}^{*}$ as functions of $\mu_{m}$. (from the paper).

(A6) $$\begin{array}{cc} \;\;\;\;\frac{\partial L}{\partial w_{i}}= & \int_{X}g\left(x,y_{i}\right)E_{i}^{*}\left(x\right)d\mu \left(x\right)+\\ \; & \;\;\;\frac{1}{\zeta }\int_{X}\sum_{j=1}^{n}g\left(x,y_{j}\right)\left(\frac{\partial \alpha^{*}\;\left(x\right)}{\partial w_{i}}+\frac{\partial \beta_{j}^{*}}{\partial w_{i}}\right)w_{j}E_{j}^{*}\left(x\right)d\mu \left(x\right). \end{array}$$

(A7) $$\begin{array}{cc} \;\;\;\;\nabla_{y_{i}}\;L= & \;\int_{X}\;\;\;\nabla_{y_{i}}g\left(x,y_{i}\right)\left(\;1-\frac{g(x,y_{i})}{\zeta }\;\right)\;E_{i}^{*}\left(x\right)w_{i}d\mu \left(x\right)+\\ \; & \;\;\frac{1}{\zeta }\;\;\int_{X}\sum_{j=1}^{n}g\left(x,y_{j}\right)\;\left(\nabla_{y_{i}}\;\alpha^{*}\;\left(x\right)\;+\;\nabla_{y_{i}}\;\beta_{j}^{*}\right)w_{j}E_{j}^{*}\left(x\right)d\mu \left(x\right). \end{array}$$

Next, we calcuate the derivatives of $\alpha^{*}$ and ${\bar{\beta }}^{*}$ by finding their defining equation and then using the Implicit Function Theorem.

The optimal solution to the dual variables $\alpha^{*}$ and $\beta^{*}$ is obtained by solving the stationary state equation $\nabla_{\alpha ,\beta }L=0$. The derivatives are taken in the sense of the Fréchet derivative. The Fenchel dual function on α and $\bar{\beta }$, has its domain and codomain $L\left(\mu ,\mu_{m};\cdot ,\cdot \right):L^{\infty }\left(X\right)\times R^{m-1}\to R$. The derivatives are

(A8) $$\begin{array}{c} \nabla_{\alpha }L\left(\mu ,\mu_{m};\alpha ,\bar{\beta }\right)=\int_{X}\left(1-\sum_{i=1}^{m}w_{i}E_{i}\left(x\right)\right)\left(\;\cdot \;\right)d\mu \left(x\right), \end{array}$$

(A9) $$\begin{array}{c} \frac{\partial }{\partial {\bar{\beta }}_{i}}L\left(\mu ,\mu_{m};\alpha ,\bar{\beta }\right)=w_{i}\left(1-\int_{X}E_{i}\left(x\right)d\mu \left(x\right)\right) \end{array}$$

where $E_{i}\left(x\right)=e^{(\alpha \left(x\right)+\beta_{i}-g\left(x,y_{i}\right))/\zeta }$ is defined as in the paper, $\nabla_{\alpha }L\left(\mu ,\mu_{m};\alpha ,\bar{\beta }\right)\in {\left(L^{\infty }\left(X\right)\right)}^{\vee }$ (as a linear functional), and $\frac{\partial }{\partial {\bar{\beta }}_{i}}L\left(\mu ,\mu_{m};\alpha ,\bar{\beta }\right)\in R$. Next, we need to show that L is differentiable in the sense of the Fréchet derivative, i.e.,

(A10) $$\begin{array}{c} \lim_{||h||\to 0}\frac{1}{\left||h|\right|}\left(L\left(\mu ,\mu_{m};\alpha +h,\bar{\beta }\right)-L\left(\mu ,\mu_{m};\alpha ,\bar{\beta }\right)\right.\left.-\nabla_{\alpha }L\left(\mu ,\mu_{m};\alpha ,\bar{\beta }\right)\left(h\right)\right)=0. \end{array}$$

By the definition of L (we write L(α) for $L(\mu ,\mu_{m};\alpha ,\bar{\beta })$),

(A11) $$\begin{array}{cc} \; & L\left(\alpha +h\right)-L\left(\alpha \right)-\nabla_{\alpha }L\left(\alpha \right)\left(h\right)\\ = & \int_{X}\;h\left(x\right)d\mu \left(x\right)\;-\;\zeta \;\int_{X}\sum_{i=1}^{m}\left(e^{h(x)/\zeta }\;-\;1\right)w_{i}E_{i}\left(x\right)d\mu \left(x\right)-\int_{X}\left(1-\sum_{i=1}^{m}w_{i}E_{i}\left(x\right)\right)h\left(x\right)d\mu \left(x\right)\\ = & \zeta \int_{X}\sum_{i=1}^{m}\left(1+\frac{h(x)}{\zeta }-e^{h(x)/\zeta }\right)E_{i}\left(x\right)w_{i}d\mu \left(x\right)\\ = & \zeta \int_{X}\left(\sum_{k=2}^{\infty }\frac{1}{k!}\frac{h{\left(x\right)}^{k}}{\zeta^{k}}\right)\sum_{i=1}^{m}E_{i}\left(x\right)w_{i}d\mu \left(x\right), \end{array}$$

The last equality is from a Taylor expansion of the exponential function. Consider that ${\left||h|\right|}_{\infty }=\mathrm{ess}\;\sup\;{|}_{x\in X}h\left(x\right)|$ the essential supremum of |h(x)| for x∈X given measure μ.

Denote $N:=\nabla_{\alpha ,\bar{\beta }}L$,

(A12) $$\begin{array}{cc} \; & \frac{1}{\left||h|\right|}\left(L\left(\alpha +h\right)-L\left(\alpha \right)-\nabla_{\alpha }L\left(\alpha \right)\left(h\right)\right)\\ \leq & \frac{\zeta }{\left||h|\right|}\int_{X}\left(\sum_{k=2}^{\infty }\frac{1}{k!}\frac{{\left|h\left(x\right)\right|}^{k}}{\zeta^{k}}\right)\sum_{i=1}^{m}E_{i}\left(x\right)w_{i}d\mu \left(x\right)\\ \leq & \frac{\zeta }{\left||h|\right|}\int_{X}\left(\sum_{k=2}^{\infty }\frac{1}{k!}\frac{{\left||h|\right|}^{k}}{\zeta^{k}}\right)\sum_{i=1}^{m}E_{i}\left(x\right)w_{i}d\mu \left(x\right)\\ = & \zeta \left(\sum_{k=2}^{\infty }\frac{1}{k!}\frac{{\left||h|\right|}^{k-1}}{\zeta^{k}}\right)\int_{X}\sum_{i=1}^{m}E_{i}\left(x\right)w_{i}d\mu \left(x\right)\\ = & \zeta \left(\sum_{k=2}^{\infty }\frac{1}{k!}\frac{{\left||h|\right|}^{k-1}}{\zeta^{k}}\right) \end{array}$$

Therefore,

(A13) $$\begin{array}{c} \lim_{||h||\to 0}\frac{1}{\left||h|\right|}\left(L\left(\alpha +h\right)-L\left(\alpha \right)\;-\;\nabla_{\alpha }L\left(\alpha \right)\left(h\right)\right)=0, \end{array}$$

which shows that the expression of $\nabla_{\alpha }L\left(\alpha \right)$ in Equation (A8) gives the correct Fréchet derivative. Note here that $\alpha \in L^{\infty }\left(X\right)$ is critical in Equation (A12).

Let $N:=\nabla_{\alpha ,\bar{\beta }}L$ values in ${\left(L^{\infty }\left(X\right)\right)}^{\vee }\times R^{m-1}$. Then, N=0 defines $\alpha^{*}$ and ${\bar{\beta }}^{*}$, which makes it possible to differentiate them about $\mu_{m}$ using the Implicit Function Theorem for Banach spaces. From now on, N take values at $\alpha =\alpha^{*}$, $\bar{\beta }={\bar{\beta }}^{*}$, i.e., the marginal conditions on $d\pi_{i}\left(x\right)=w_{i}E_{i}\left(x\right)d\mu \left(x\right)$ hold.

Thus, we need $\nabla_{\alpha ,\bar{\beta }}N$ and $\nabla_{w_{i},y_{i}}N$ calculated, and prove that $\nabla_{\alpha ,\bar{\beta }}N$ is invertible (and give the inverse).

It is necessary to make sure which form $\nabla_{\alpha ,\bar{\beta }}N$ is in according to the Fréchet derivative. Start from the map $N\left(\mu ,\mu_{m};\cdot ,\cdot \right):\left(L^{\infty }\left(X\right)\right)\times R^{m-1}\to {\left(L^{\infty }\left(X\right)\right)}^{\vee }\times {\left(R^{m-1}\right)}^{\vee }$, where $R^{m-1}$ is isomorphic to its dual Banach space ${\left(R^{m-1}\right)}^{\vee }$. Then, $\nabla_{\alpha ,\bar{\beta }}N\in {\mathrm{Hom}}_{R}^{b}\left(L^{\infty }\left(X\right)\times R^{m-1},{\left(L^{\infty }\left(X\right)\right)}^{\vee }\times {\left(R^{m-1}\right)}^{\vee }\right)$, where ${\mathrm{Hom}}^{b}$ represents the set of bounded linear operators. Moreover, recall that (·)⊗A is the left adjoint functor of ${\mathrm{Hom}}_{R}^{b}\left(A,\cdot \right)$; then, for R-vector spaces, ${\mathrm{Hom}}_{R}^{b}\left(L^{\infty }\left(X\right)\times R^{m-1},{\left(L^{\infty }\left(X\right)\right)}^{\vee }\times {\left(R^{m-1}\right)}^{\vee }\right)≅{\mathrm{Hom}}_{R}^{b}\left({\left(L^{\infty }\left(X\right)\times R^{m-1}\right)}^{⊗2},R\right)$. Thus, we can write $\nabla_{\alpha ,\bar{\beta }}N$ in terms of a bilinear form on vector space $L^{\infty }\left(X\right)\times R^{m-1}$.

From the expression of N, we may differentiate (similarly as the calculations (A11) to (A13)):

(A14) $$\begin{array}{c} \nabla_{\alpha }N=\left(-\frac{1}{\zeta }\int_{X}\left(\cdot \right)\left(-\right)\sum_{i=1}^{m}w_{i}E_{i}\left(x\right)d\mu \left(x\right)\right.,\left.-\frac{1}{\zeta }\int_{X}\left(\cdot \right)w_{i}E_{i}\left(x\right)d\mu \left(x\right)\right) \end{array}$$

(A15) $$\begin{array}{c} \nabla_{\bar{\beta }}N=\left(-\frac{1}{\zeta }\int_{X}\left(\cdot \right)w_{i}E_{i}\left(x\right)d\mu \left(x\right)\right.,\left.-\frac{\delta_{ij}}{\zeta }\int_{X}w_{i}E_{i}\left(x\right)d\mu \left(x\right)\right) \end{array}$$

Consider the boundary conditions $\sum_{i=1}^{m}w_{i}E_{i}\left(x\right)d\mu \left(x\right)=\sum_{i=1}^{m}d\pi_{i}\left(x\right)=\mu \left(x\right)$ and $\int_{X}w_{i}E_{i}\left(x\right)d\mu \left(x\right)=\int_{X}\pi_{i}\left(x\right)=w_{i}$. The $\nabla_{\alpha ,\bar{\beta }}N$ as the Hessian form of L can be written as

(A16) $$\begin{array}{c} \nabla_{\alpha ,\bar{\beta }}N=-\frac{1}{\zeta }\left[\begin{array}{cc} \langle -,\cdot \rangle & \langle \pi_{j},\cdot \rangle \\ \langle -,\pi_{i}\rangle & w_{i}\delta_{ij} \end{array}\right] \end{array}$$

with $\langle \phi_{1},\phi_{2}\rangle =\int_{X}\phi_{1}\left(x\right)\phi_{2}\left(x\right)\mu \left(x\right)$, or further as

(A17) $$\begin{array}{c} \nabla_{\alpha ,\bar{\beta }}N=-\frac{1}{\zeta }\left[\begin{array}{cc} d\mu \left(x\right)d\mu \left(x^{\prime }\right)\delta \left(x,x^{\prime }\right) & d\pi_{j}\left(x\right)\\ d\pi_{i}\left(x^{\prime }\right) & w_{i}\delta_{ij} \end{array}\right] \end{array}$$

over the basis ${\left\{\delta \left(x\right),e_{i}\right\}}_{x\in X,i<m}$.

By the inverse of $\nabla_{\alpha ,\bar{\beta }}N$, we mean the element in ${\mathrm{Hom}}_{R}^{b}\left({\left(L^{\infty }\left(X\right)\right)}^{\vee }\times {\left(R^{m-1}\right)}^{\vee },\right.$ $\left.L^{\infty }\left(X\right)\times R^{m-1}\right)$ which composes with $\nabla_{\alpha ,\bar{\beta }}N$ (on the left and on the right) as identities. By the natural identity between double dual $V^{\vee \vee }≅V$ and the tensor hom adjunction,

(A18) $$\begin{array}{cc} \; & {\mathrm{Hom}}_{R}^{b}\left({\left(L^{\infty }\left(X\right)\right)}^{\vee }\;\times \;{\left(R^{m-1}\right)}^{\vee },L^{\infty }\left(X\right)\times R^{m-1}\right)\\ ≅ & {\mathrm{Hom}}_{R}^{b}\left({\left(L^{\infty }\left(X\right)\right)}^{\vee }\;\times \;{\left(R^{m-1}\right)}^{\vee },{\left(L^{\infty }\left(X\right)\;\times \;R^{m-1}\right)}^{\vee \vee }\right)\\ ≅ & {\mathrm{Hom}}_{R}^{b}\left({\left({\left(L^{\infty }\left(X\right)\right)}^{\vee }\times {\left(R^{m-1}\right)}^{\vee }\right)}^{⊗2},R\right), \end{array}$$

we can write the inverse of $\nabla_{\alpha ,\bar{\beta }}N$ as a bilinear form again.

Denote $\nabla_{\alpha ,\bar{\beta }}N$ in the block form $\left[\begin{array}{cc} A & B\\ B^{T} & D \end{array}\right]$. According to the block-inverse formula

(A19) $${\left[\begin{array}{cc} A & B\\ B^{T} & D \end{array}\right]}^{-1}\;\;=\;\left[\;\begin{array}{cc} A^{-1}\;\;+\;A^{-1}BF^{-1}B^{T}A^{-1} & -A^{-1}BF^{-1}\\ -F^{-1}B^{T}A^{-1} & F^{-1} \end{array}\;\right],$$

where $F=D-B^{T}A^{-1}B$, whose invertibility determines the invertibility of $\left[\begin{array}{cc} A & B\\ B^{T} & D \end{array}\right]$.

Consider that $A^{-1}\in {\mathrm{Hom}}_{R}^{b}\left({\left(L^{\infty }{\left(X\right)}^{\vee }\right)}^{⊗2},R\right)$; explicitly, $A^{-1}\left(x,y\right)=\delta \left(x,y\right)$. Therefore, from Equation (A17),

(A20) $$\begin{array}{cc} F_{ij}= & \delta_{ij}w_{i}-\int_{X^{2}}\delta \left(x,x^{\prime }\right)\pi_{i}\left(x\right)\pi_{j}\left(x^{\prime }\right)\\ = & \delta_{ij}w_{i}-\int_{X}w_{i}w_{j}E_{i}\left(x\right)E_{j}\left(x\right)\mu \left(x\right). \end{array}$$

The matrix *F* is symmetric, of rank m−1, and strictly diagonally dominant; therefore, it is invertible. To see the strictly diagonal dominance, consider $\sum_{j=1}^{m}\int_{X}w_{i}w_{j}E_{i}\left(x\right)E_{j}\left(x\right)\mu \left(x\right)=\int_{X}w_{i}E_{i}\left(x\right)\sum_{j=1}^{m}w_{j}E_{j}\left(x\right)\mu \left(x\right)=\int_{X}w_{i}E_{i}\left(x\right)\mu \left(x\right)=w_{i}$ by applying the marginal conditions. The matrix *F* is of size (m−1)×(m−1) (there is no i=m or j=m for $F_{ij}$). Then, the matrix *F* is strictly diagonally dominant.

With all ingradients known in formula (A19), we can calculate the inverse of $\nabla_{\alpha ,\bar{\beta }}N$.

Following the implicit function theorem, we need $\nabla_{w_{i},y_{i}}N$; each partial derivative is an element in $L^{\infty }{\left(X\right)}^{\vee }\times R^{m-1}$.

(A21) $$\begin{array}{cc} \frac{\partial N}{\partial w_{i}}= & \left(-\int_{X}E_{i}\left(x\right)\left(\cdot \right)d\mu \left(x\right),\delta_{ij}\left(1-\int_{X}E_{i}\left(x\right)d\mu \left(x\right)\right)\right)\\ = & \left(-\int_{X}\left(\cdot \right)E_{i}\left(x\right)d\mu \left(x\right),0\right). \end{array}$$

Note that if we apply the constraint $\sum_{i=1}^{m}w_{i}=1$ to the $w_{i}$s, we may set $w_{m}=1-\sum_{i=1}^{m-1}w_{i}$ and recalculate the above derivatives as $\nabla_{w_{i}^{\prime }}N=\nabla_{w_{i}}N-\nabla_{w_{m}}N$ when i≠m and $\nabla_{w_{m}^{\prime }}N=\sum_{i=1}^{m-1}\nabla_{w_{i}}N$.

(A22) $$\begin{array}{cc} \nabla_{y_{i}}N= & \left(\frac{1}{\zeta }\int_{X}\left(\cdot \right)\nabla_{y_{i}}g\left(x,y_{i}\right)w_{i}E_{i}\left(x\right)d\mu \left(x\right),\right.\left.\frac{\delta_{ij}}{\zeta }\int_{X}\nabla_{y_{i}}g\left(x,y_{i}\right)w_{i}E_{i}\left(x\right)d\mu \left(x\right)\right) \end{array}$$

Finally, by the Implicit Function Theorem,

$$\nabla_{w_{i},y_{j}}\left(\alpha^{*},{\bar{\beta }}^{*}\right)=-{\left(\nabla_{\alpha ,\bar{\beta }}{N|}_{\alpha^{*},{\bar{\beta }}^{*}}\right)}^{-1}\left(\nabla_{w_{i},y_{j}}{N|}_{\alpha^{*},{\bar{\beta }}^{*}}\right)$$

which fits in (A6) and (A7).

### Appendix B.2. Second Derivatives

In this part, we calculate the second derivatives of $W_{k,\zeta }^{k}\left(\mu ,\mu_{m}\right)$ with respect to the ingredients of $\mu_{m}$, i.e., $w_{i}$s and $y_{i}$s, for the potential of applying Newton’s method to the EDOT (which we have not implemented yet).

Using the previous results, we can further calculate the second derivatives of $W_{k,\zeta }^{k}$ about $w_{i}$s and $y_{i}$s. Differentiating (A6) and (A7) results in

(A23) $$\begin{array}{cc} \frac{\partial^{2}L}{\partial w_{i}\partial w_{j}}= & \frac{1}{\zeta }\int_{X}g\left(x,y_{j}\right)\sum_{k=i,j}\left(\frac{\partial \alpha (x)}{\partial w_{k}}+\frac{\partial {\bar{\beta }}_{k}}{\partial w_{k}}\right)E_{k}\left(x\right)d\mu \left(x\right)\\ \; & +\frac{1}{\zeta }\int_{X}\sum_{k=1}^{n}\left(\frac{\partial^{2}\alpha \left(x\right)}{\partial w_{i}\partial w_{j}}+\frac{\partial^{2}{\bar{\beta }}_{k}}{\partial w_{i}\partial w_{j}}\right)w_{k}E_{k}\left(x\right)d\mu \left(x\right)\\ \; & +\frac{1}{\zeta^{2}}\int_{X}\sum_{k=1}^{m}\prod_{l=i,j}\left(\frac{\partial \alpha (x)}{\partial w_{l}}+\frac{\partial {\bar{\beta }}_{k}}{\partial w_{l}}\right)w_{k}E_{k}\left(x\right)d\mu \left(x\right) \end{array}$$

(A24) $$\begin{array}{cc} \nabla_{y_{j}}\frac{\partial L}{\partial w_{i}}= & \delta_{ij}\left[\int_{X}\left(1-\frac{g(x,y_{i})}{\zeta }\right)\nabla_{y_{i}}E_{i}\left(x\right)d\mu \left(x\right)\right]\\ \; & +\frac{1}{\zeta }\int_{X}\nabla_{y_{j}}g\left(x,y_{j}\right)\left(\frac{\partial \alpha (x)}{\partial w_{i}}+\frac{\partial {\bar{\beta }}_{j}}{\partial w_{i}}\right)w_{j}E_{j}\left(x\right)d\mu \left(x\right)\\ \; & +\frac{1}{\zeta }\int_{X}g\left(x,y_{j}\right)\nabla_{y_{j}}\left(\frac{\partial \alpha (x)}{\partial w_{i}}+\frac{\partial {\bar{\beta }}_{j}}{\partial w_{i}}\right)w_{j}E_{j}\left(x\right)d\mu \left(x\right)\\ \; & +\frac{1}{\zeta^{2}}\int_{X}\sum_{k=1}^{m}g\left(x,y_{k}\right)\left(\frac{\partial \alpha (x)}{\partial w_{i}}+\frac{\partial {\bar{\beta }}_{k}}{\partial w_{i}}\right)\left(\nabla_{y_{j}}\alpha \left(x\right)+\nabla_{y_{j}}{\bar{\beta }}_{k}\right)w_{k}E_{k}\left(x\right)d\mu \left(x\right) \end{array}$$

(A25) $$\begin{array}{cc} \nabla_{y_{j}}\nabla_{y_{i}}L= & \delta_{ij}\left[\int_{X}\nabla_{y_{i}}^{2}g\left(x,y_{i}\right)\left(1-\frac{g(x,y_{i})}{\zeta }\right)w_{i}E_{i}\left(x\right)d\mu \left(x\right)\right.\\ \; & \left.-\frac{1}{\zeta }\int_{X}{\left(\nabla_{y_{i}}g\left(x,y_{i}\right)\right)}^{2}\left(2-\frac{g(x,y_{i})}{\zeta }\right)w_{i}E_{i}\left(x\right)d\mu \left(x\right)\right]\\ \; & +\frac{1}{\zeta }\int_{X}\left(1-\frac{g(x,y_{i})}{\zeta }\right)\nabla_{y_{i}}g\left(x,y_{i}\right)\cdot \left(\nabla_{y_{j}}\alpha \left(x\right)+\nabla_{y_{j}}\bar{\beta_{i}}\right)w_{i}E_{i}\left(x\right)d\mu \left(x\right)\\ \; & +\frac{1}{\zeta }\int_{X}\left(1-\frac{g(x,y_{j})}{\zeta }\right)\nabla_{y_{j}}g\left(x,y_{j}\right)\cdot \left(\nabla_{y_{i}}\alpha \left(x\right)+\nabla_{y_{i}}\bar{\beta_{j}}\right)w_{j}E_{j}\left(x\right)d\mu \left(x\right)\\ \; & +\frac{1}{\zeta }\int_{X}\sum_{k=1}^{m}g\left(x,y_{k}\right)\left(\nabla_{y_{i}}\nabla_{y_{j}}\alpha \left(x\right)+\nabla_{y_{i}}\nabla_{y_{j}}{\bar{\beta }}_{k}\right)\cdot w_{k}E_{k}\left(x\right)d\mu \left(x\right)\\ \; & +\frac{1}{\zeta^{2}}\int_{X}\sum_{k=1}^{m}g\left(x,y_{k}\right)\prod_{l=i,j}\left(\nabla_{l}\alpha +\nabla_{l}{\bar{\beta }}_{k}\right)\cdot w_{k}E_{k}\left(x\right)d\mu \left(x\right) \end{array}$$

Once we have the second derivatives of g(x,y) on $y_{i}$s, we need the second derivatives of $\alpha^{*}$ and ${\bar{\beta }}^{*}$ to build the above second derivatives. From the formula $\nabla_{w_{i},y_{j}}\left(\alpha^{*},{\bar{\beta }}^{*}\right)=-{\left(\nabla_{\alpha ,\bar{\beta }}{N|}_{\alpha^{*},{\bar{\beta }}^{*}}\right)}^{-1}\left(\nabla_{w_{i},y_{j}}{N|}_{\alpha^{*},{\bar{\beta }}^{*}}\right)$, we can differentiate

(A26) $$\begin{array}{cc} \; & \nabla_{w_{k},y_{l}}\nabla_{w_{i},y_{j}}\left(\alpha^{*},{\bar{\beta }}^{*}\right)\\ = & -\nabla_{w_{k},y_{l}}{\left(\nabla_{\alpha ,\bar{\beta }}{N|}_{\alpha^{*},{\bar{\beta }}^{*}}\right)}^{-1}\left(\nabla_{w_{i},y_{j}}{N|}_{\alpha^{*},{\bar{\beta }}^{*}}\right)-{\left(\nabla_{\alpha ,\bar{\beta }}{N|}_{\alpha^{*},{\bar{\beta }}^{*}}\right)}^{-1}\left(\nabla_{w_{k},y_{l}}\nabla_{w_{i},y_{j}}{N|}_{\alpha^{*},{\bar{\beta }}^{*}}\right). \end{array}$$

Here, from the formula that $\nabla A^{-1}=-A^{-1}\nabla AA^{-1}$ (this is the product rule for $AA^{-1}=I$), we have

(A27) $$\begin{array}{c} \nabla_{w_{k},y_{l}}{\left(\nabla_{\alpha ,\bar{\beta }}{N|}_{\alpha^{*},{\bar{\beta }}^{*}}\right)}^{-1}=-{\left(\nabla_{\alpha ,\bar{\beta }}{N|}_{\alpha^{*},{\bar{\beta }}^{*}}\right)}^{-1}\nabla_{w_{k},y_{l}}\left(\nabla_{\alpha ,\bar{\beta }}{N|}_{\alpha^{*},{\bar{\beta }}^{*}}\right)\;{\left(\nabla_{\alpha ,\bar{\beta }}{N|}_{\alpha^{*},{\bar{\beta }}^{*}}\right)}^{-1} \end{array}$$

and

(A28) $$\begin{array}{c} \nabla_{w_{k}}\left(\nabla_{\alpha ,\bar{\beta }}{N|}_{\alpha^{*},{\bar{\beta }}^{*}}\right)=-\frac{1}{\zeta }\left[\begin{array}{cc} 0 & \delta_{jk}E_{k}\left(x\right)d\mu \left(x\right)\\ \delta_{ik}E_{k}\left(x^{\prime }\right)d\mu \left(x^{\prime }\right) & \delta_{ij}\delta_{jk} \end{array}\right] \end{array}$$

(A29) $$\begin{array}{c} \nabla_{y_{k}}\left(\nabla_{\alpha ,\bar{\beta }}{N|}_{\alpha^{*},{\bar{\beta }}^{*}}\right)=\frac{1}{\zeta^{2}}\left[\begin{array}{cc} 0 & \delta_{jk}\nabla_{y_{k}}g\left(x,y_{k}\right)d\pi_{k}\left(x\right)\\ \delta_{ik}\nabla_{y_{k}}g\left(x^{\prime },y_{k}\right)d\pi_{k}\left(x^{\prime }\right) & 0 \end{array}\right]. \end{array}$$

The last piece we need is $\left(\nabla_{w_{k},y_{l}}\nabla_{w_{i},y_{j}}{N|}_{\alpha^{*},{\bar{\beta }}^{*}}\right)$:

(A30) $$\begin{array}{c} \frac{\partial^{2}N}{\partial w_{j}\partial w_{i}}=\left(0,0\right), \end{array}$$

(A31) $$\begin{array}{c} \nabla_{y_{j}}\frac{\partial N}{\partial w_{i}}=\left(\frac{1}{\zeta }\int_{X}\left(\cdot \right)\nabla_{y_{j}}g\left(x,y_{j}\right)E_{i}\left(x\right)d\mu \left(x\right),0\right), \end{array}$$

(A32) $$\begin{array}{cc} \nabla_{y_{j}}\nabla_{y_{i}}N= & \frac{\delta_{ij}}{\zeta }\left(\int_{X}\left(\cdot \right)\nabla_{y_{i}}^{2}g\left(x,y_{i}\right)w_{i}E_{i}\left(x\right)d\mu \left(x\right)\right.+\int_{X}\left(\cdot \right){\left(\nabla_{y_{i}}g\left(x,y_{i}\right)\right)}^{2}w_{i}E_{i}\left(x\right)d\mu \left(x\right),\\ \; & \delta_{ik}\int_{X}\nabla_{y_{i}}^{2}g\left(x,y_{i}\right)w_{i}E_{i}\left(x\right)d\mu \left(x\right)+\left.\int_{X}{\left(\nabla_{y_{i}}g\left(x,y_{i}\right)\right)}^{2}w_{i}E_{i}\left(x\right)d\mu \left(x\right)\right), \end{array}$$

where in the last one, *k*, represents the *k*-th component in N’s second part (about $\bar{\beta }$).

## Appendix C. Algorithms

### Appendix C.1. Algorithm: Simple EDOT

The following states the Algorithm A1 of Simple EDOT.

**Algorithm A1** Simple EDOT using minibatch SGD
1: **input:** μ, *k*, *m*, ζ, *N* batch size, ϵ for stopping criterion, α=0.2 for momentum, β=0.2 for learning rate. 2: **output:** $x_{i}\in X$, $w_{i}>0$ such that $\mu_{m}=\sum_{i=1}^{m}w_{i}\delta_{x_{i}}$. 3: **initialize:** randomly choose ∑i=1mwi(0)δxi(0)|; set t=0; set learning rate $\eta_{t}=0.5{\left(1+\beta t\right)}^{-s}$ (for t>0 and s∈(0.5,1]). 4: **repeat** 5: Set t←t+1; 6: Sample *N* points ${\left\{y_{j}\right\}}_{j=1}^{N}\subseteq X$ from μ independently; 7: Construct $\mu_{N}^{\left(t\right)}=\frac{1}{N}\sum_{j=1}^{N}\delta_{y_{j}}$; 8: Calculate gradients $\nabla_{x}{\hat{W}}_{k,\zeta }^{k}\left(\mu_{N}^{\left(t\right)},\mu_{m}^{\left(t\right)}\right)$ and $\nabla_{w}{\hat{W}}_{k,\zeta }^{k}\left(\mu_{N}^{\left(t\right)},\mu_{m}^{\left(t\right)}\right)$; 9: Update $Dx_{t}\leftarrow \alpha Dx_{t-1}+\nabla_{x}{\hat{W}}_{k,\zeta }^{k}\left(\mu_{N}^{\left(t\right)},\mu_{m}^{\left(t\right)}\right)$, $Dw_{t}\leftarrow \alpha Dw_{t-1}+\nabla_{w}{\hat{W}}_{k,\zeta }^{k}\left(\mu_{N}^{\left(t\right)},\mu_{m}^{\left(t\right)}\right)$; 10: Update $x^{\left(t\right)}\leftarrow x^{\left(t-1\right)}-\eta_{t}Dx_{t}$, $w^{\left(t\right)}\leftarrow w^{\left(t-1\right)}-\eta_{t}Dw_{t}$; 11: **until**$|\nabla_{x}{\hat{W}}_{k,\zeta }^{k}\left|+\right|\nabla_{w}{\hat{W}}_{k,\zeta }^{k}|<\epsilon$; 12: Set output $x_{i}\leftarrow x_{i}^{\left(t\right)}$, $w_{i}\leftarrow w_{i}^{\left(t\right)}$.

### Appendix C.2. Proof of Proposition 2

**Remark** **A1.**
*The convergence to a stationary point in expectation means that the liminf of the expected norm of the gradient over all the sequences considered approaches to 0.*

**Proof.** Discrete distributions of size *m* can be parameterized by $X^{m}\times \Delta$ in terms of $\sum_{i=1}^{m}p_{i}\delta_{y_{i}}$ with $(p_{1},p_{2},\cdots ,p_{m})\in \Delta$ and $y_{i}\in X$. To make the SGD work, we assume that *X* is a path-connected subset of $R^{d}$ of dimension *d*. For ϵ>0, let $\Delta_{\epsilon }=\Delta_{\epsilon }^{m-1}=\left\{\left(p_{1},p_{2},\cdots ,p_{m}\right):\sum_{i=1}^{m}p_{i}=1,p_{i}\geq \epsilon \right\}$. First, we prove the claim by assuming that (*) the set of limit points of $\left(\mu_{m}^{\left(i\right)}\right)$ is contained in $X^{m}\times \Delta_{\epsilon }$. According to Theorem 4.10 and Corollary 4.12 of [25], to show that Algorithm A1 converges to a stationary point in expectation, i.e., ${lim inf}_{i\to 0}E[||\nabla W_{k,\zeta }^{k}\left(\mu ,\mu_{m}^{\left(i\right)}\right){\left|\right|}^{2}]=0$, one needs to check: (1). $W_{k,\zeta }^{k}\left(\mu ,\mu_{m}^{\left(i\right)}\right)$ is second-differentiable; (2). $\nabla W_{k,\zeta }^{k}\left(\mu ,\mu_{m}^{\left(i\right)}\right)$ is Lipschitz continuous; and (3). $E[||\nabla {\hat{W}}_{k,\zeta }^{k}\left(\mu_{N}^{\left(i\right)},\mu_{m}^{\left(i\right)}\right)-\nabla W_{k,\zeta }^{k}\left(\mu ,\mu_{m}^{\left(i\right)}\right)|{|}^{2}]$ is bounded. (1). The second differentiablity of $W_{k,\zeta }^{k}\left(\mu ,\mu_{m}^{\left(i\right)}\right)$ is shown in Appendix B.2 with the second derivative calculated. (2). As a consequence of (a), we have that $\nabla W_{k,\zeta }^{k}\left(\mu ,\mu_{m}^{\left(i\right)}\right)$ is continuous. Moreover, by checking each factor of $\nabla^{2}W_{k,\zeta }^{k}\left(\mu ,\mu_{m}^{\left(i\right)}\right)$ shown in Appendix B.2, we can see that $\nabla^{2}W_{k,\zeta }^{k}\left(\mu ,\mu_{m}^{\left(i\right)}\right)$ is bounded. (Actually, we need $\det(\nabla_{\alpha ,\bar{\beta }}N)$ finite to make the SIM bounded, which is true in $\Delta_{\epsilon }$.) Therefore, $\nabla W_{k,\zeta }^{k}\left(\mu ,\mu_{m}^{\left(i\right)}\right)$ is Lipschitz continuous. (3). Noise has bounded variance: Equivalently, we just need to check that $\mathrm{Var}(||\nabla W_{k,\zeta }^{k}\left(\mu_{N},\mu_{m}\right)|{|}^{2})$ is finite, where $\mu_{N}$ is the empirical distribution with *N* samples taken (which is stochastic), and $\mu_{m}$ is the fixed discretization in $X^{m}\times \Delta_{\epsilon }$ (this need not to be the “optimal” one). $\nabla W_{k,\zeta }^{k}\left(\mu_{N}^{\left(i\right)},\mu_{m}^{\left(i\right)}\right)$ is continuous with respect to both $\mu_{N}^{\left(i\right)}$ and $\mu_{m}^{\left(i\right)}$; hence, it is continuous over compact space $X^{N}\times X^{m}\times \Delta_{\epsilon }$; hence, it is bounded by a constant *C*. Thus, the proposition holds with assumption (∗). Further suppose that assumption (∗) does not hold. Then, for any sequence $\epsilon_{1},\epsilon_{2}\cdots \to 0$, there always exist infinite limit points of $\left(\mu_{m}^{\left(i\right)}\right)$ that lie outside $\Delta_{\epsilon_{k}}$ for any k>0. Therefore, we can construct a subsequence of $\mu_{m}^{\left(i\right)}$ converging to a point $p\in X^{m}\times \partial \Delta$. Thus, *p* is also a limit point. This contradicts the assumption that the set of limit points of $\left(\mu_{m}^{\left(i\right)}\right)$ does not intersect with $X^{m}\times \partial \Delta$. The proof is then complete.    □

### Appendix C.3. Proof of Proposition 3

**Proof.** First, for each iteration in the minibatch SGD, let *N* be the sample (minibatch) size of $\mu_{N}$ for approximating μ. Let *m* be the size of target discretization $\mu_{m}$ (the output). Furthermore, let *d* be the dimension of *X* and ϵ be the error bound in the Sinkhorn calculation for the entropy-regularized optimal transference plan between $\mu_{N}$ and $\mu_{m}$. The Sinkhorn algorithm for the positive matrix $e^{-g(x,y)/\zeta }$ (of size N×m) converges linearly, which takes O(log(1/ϵ)) steps to fall into a region of radius ϵ, thus contributing O(Nmlog(1/ϵ)) in time complexity. The inverse matrix M of $\nabla_{\left(\alpha ,\bar{\beta }\right)}N=-\frac{1}{\zeta }\left[\begin{array}{cc} A & B\\ B^{T} & D \end{array}\right]$ (Equation (6)) is taken block-wise

$$M=-\zeta \left[\begin{array}{cc} A^{-1}+A^{-1}BE^{-1}B^{T}A^{-1} & -A^{-1}BE^{-1}\\ -E^{-1}B^{T}A^{-1} & E^{-1} \end{array}\right],$$

where $E=D-B^{T}A^{-1}B$. Block *E* is constructed in $O(Nm^{2})$ and inverted in $O(m^{3})$; block $A^{-1}BE^{-1}$ takes $O(Nm^{2})$, as *A* is diagonal; and the block $A^{-1}+A^{-1}BE^{-1}B^{T}A^{-1}$ takes $O(N^{2}m)$ to construct. When m≪N, the time complexity in constructing M is $O(N^{2}m)$. From M to the gradient of dual variables, the tensor contractions have complexity $O({\left(N+m\right)}^{2}md)$. Finally, to get the gradient, the complexity is dominated by the second term of $\nabla_{y_{i}}\;W_{k,\zeta }^{k}$ (see Equation (5)), which is a contraction between a matrix Nm (i.e., gdπ(x)) with tensors of sizes Nmd and $m^{2}d$ (two gradients on the dual variables α and β) along *N* and *m*, respectively. Thus, the final step contributes O((N+m)md). The time complexity of increment steps in the SGD turns out to be O(md). Therefore, for *L* steps of the minibatch SGD, the time complexity is $O({\left(N+m\right)}^{2}mdL+NmL\log\left(1/\epsilon \right))$. For space complexity of the simple EDOT, the Sinkhorn algorithm (which can be done in position O(Nm)) is the only iterative computation in a single SGD step, and between two SGD steps, only the resulting distribution is passed to the next step. Therefore, the space complexity is $O({\left(N+m\right)}^{2})$ coming from the M; others are, at most, of size O(m(N+m)).    □

### Appendix C.4. Adaptive Refinement via DFS: Midpoints

The pseudocode for the division algorithm of the adaptive EDOT using KD-tree refinement cutting at the midpoints is shown in Algorithm A2. The $Round_{0.5↑}$ means the rounding method with 0.5 rounded up, and $Round_{0.5↓}$ is that with 0.5 rounded down; thus, the discretization point is correctly partitioned.

**Algorithm A2** Adaptive Refinement via Depth First Search
1: **input:** *m*, μ, $N_{0}$ sample size, $m_{*}$ max number of points in a cell, $a=(a_{0},a_{1},\cdots ,a_{d-1})$ and $b=(b_{0},b_{1},\cdots ,b_{d-1})$ as lower/upper bounds of the region; 2: **output:** out: stack of subproblems $\left(S_{i},m_{i},p_{i}\right)$ with $\sum p_{i}=1$, $\sum m_{i}=m$, $\sum |S_{i}|=N_{0}$; 3: **initialization:** $p_{0}\leftarrow 1$; *T*, out be empty stacks; 4: Sample $N_{0}$ points $S_{0}∼\mu$; 5: *T*.push(($S_{0}$, *m*, $p_{0}$, a, b)); 6: **while** *T* is not empty **do** 7: (*S*, *m*, *p*, a, b) ←*T*.pop(); 8: $l\leftarrow \mathrm{argmax}\;\{b_{i}-a_{i}\}$, $mid\leftarrow (a_{l}+b_{l})/2$; 9: $a^{\left(1\right)}\leftarrow a$, $a^{\left(2\right)}\leftarrow \left(a_{0},\cdots ,mid,\cdots ,a_{d-1}\right)$; 10: $b^{\left(1\right)}\leftarrow \left(b_{0},\cdots ,mid,\cdots ,b_{d-1}\right)$, $b^{\left(2\right)}\leftarrow b$; 11: $S_{1}\leftarrow \left\{x\in S:x_{l}<=mid\right\}$, $S_{2}\leftarrow \left\{x\in S:x_{l}>mid\right\}$, $N_{1}\leftarrow \left|S_{1}\right|$, $N_{2}\leftarrow \left|S_{2}\right|$; 12: $m_{1}\leftarrow Round_{0.5↑}\left(\frac{N_{1}^{d/(k+d)}}{N_{1}^{d/(k+d)}+N_{2}^{d/(k+d)}}\right)$; 13: $m_{2}\leftarrow Round_{0.5↓}\left(\frac{N_{2}^{d/(k+d)}}{N_{1}^{d/(k+d)}+N_{2}^{d/(k+d)}}\right)$; 14: **if** $m_{1}=0$ **then** 15: $p_{1}\leftarrow 0$, $p_{2}\leftarrow \left(N_{1}+N_{2}\right)/N_{0}$; 16: **else if** $m_{2}=0$ **then** 17: $p_{1}\leftarrow \left(N_{1}+N_{2}\right)/N_{0}$, $p_{2}\leftarrow 0$; 18: **else** 19: $p_{1}\leftarrow N_{1}/N_{0}$, $p_{2}\leftarrow N_{2}/N_{0}$; 20: **end if** 21: **for** i←1,2 **do** 22: **if** $m_{i}>m_{*}$ **then** 23: *T*.push($\left(S_{i},m_{i},p_{i},a^{\left(i\right)},b^{\left(i\right)}\right)$); 24: **else if** $m_{i}>0$ **then** 25: out.push($\left(S_{i},m_{i},p_{i}\right)$); 26: **end if** 27: **end for** 28: **end while**

**Algorithm A3** Adaptive EDOT Variation 1
1: **input:** *m*, μ, $N_{0}$ sample size, $m_{*}$ max number of points in a cell, $a=(a_{0},a_{1},\cdots ,a_{d-1})$ and $b=(b_{0},b_{1},\cdots ,b_{d-1})$ as lower/upper bounds of the region, let *R* be resolution for occupied volume; 2: **output:** out: stack of subproblems $\left(S_{i},m_{i},p_{i}\right)$ with $\sum p_{i}=1$, $\sum m_{i}=m$; 3: **initialization:** $p_{0}\leftarrow 1$; 4: **procedure** BuildTree(node) 5: a←node.lower, b←node.upper 6: $l\leftarrow \mathrm{argmax}\;\{b_{i}-a_{i}\}$, $mid\leftarrow (a_{l}+b_{l})/2$; 7: $a^{\left(0\right)}\leftarrow a$, $a^{\left(1\right)}\leftarrow \left(a_{0},\cdots ,a_{l-1},mid,a_{l+1}\cdots ,a_{d-1}\right)$; 8: $b^{\left(0\right)}\leftarrow \left(b_{0},\cdots ,b_{l-1},mid,\cdots ,b_{l+1},b_{d-1}\right)$, $b^{\left(1\right)}\leftarrow b$; 9: $S_{0}\leftarrow \left\{x\in S:x_{l}<=mid\right\}$, $S_{1}\leftarrow \left\{x\in S:x_{l}>mid\right\}$; 10: **for** i←0,1; **do** 11: **if** $\prod_{j=0}^{d-1}\left(b_{j}-a_{j}\right)>R$ and $|S_{i}|>0$; **then** 12: $node.child\left[i\right]=\{occVol:0,lower:a^{\left(i\right)},upper:b^{\left(i\right)},sample:S_{i},child:Array\left(2\right),discSize:0\}$; 13: BuildTree(node.child[i]) 14: **else if** $|S_{i}|=0$; **then** 15: $node.child\left[i\right]=\{occVol:0,lower:a^{\left(i\right)},upper:b^{\left(i\right)},sample:S_{i},child:Array\left(2\right),discSize:0\}$ 16: **else** 17: $node.child\left[i\right]=\{occVol:\prod_{j=0}^{d-1}\left(b_{j}^{\left(i\right)}-a_{j}^{\left(i\right)}\right),lower:a^{\left(i\right)},upper:b^{\left(i\right)},sample:S_{i},child:Array\left(2\right),discSize:0\}$ 18: **end if** 19: **end for** 20: node.occVol←node.child[0].occVol+node.child[1].occVol; 21: **end procedure** 22: Sample $N_{0}$ points $S_{0}∼\mu$; 23: $Root=\{occVol:0,lower:a,upper:b,sample:S_{0},child:Array\left(2\right),discSize:0\}$; 24: BuildTree(Root); 25: T,out← empty stack; 26: T.push(Root) 27: **while** *T* is not empty **do** 28: node←T.pop(); 29: $P_{0}\leftarrow \left|node.child\left[0\right].sample\right|$, $P_{1}\leftarrow \left|node.child\left[1\right].sample\right|$; 30: $V_{0}\leftarrow node.child\left[0\right].occVol$, $V_{1}\leftarrow node.child\left[1\right].occVol$; 31: $M_{0}\leftarrow P_{0}\cdot V_{0}^{k/d}$, $M_{1}\leftarrow P_{1}\cdot V_{1}^{k/d}$; 32: $m_{0}\leftarrow Round_{0.5↑}\left(\frac{M_{0}^{d/(k+d)}}{M_{0}^{d/(k+d)}+M_{1}^{d/(k+d)}}\right)$; 33: $m_{1}\leftarrow Round_{0.5↓}\left(\frac{M_{1}^{d/(k+d)}}{M_{0}^{d/(k+d)}+M_{1}^{d/(k+d)}}\right)$; 34: **if** $m_{0}=0$ **then** 35: $p_{0}\leftarrow 0$, $p_{1}\leftarrow \left(M_{0}+M_{1}\right)/N_{0}$; 36: **else if** $m_{1}=0$ **then** 37: $p_{0}\leftarrow \left(M_{0}+M_{1}\right)/N_{0}$, $p_{1}\leftarrow 0$; 38: **else** 39: $p_{0}\leftarrow M_{0}/N_{0}$, $p_{1}\leftarrow M_{1}/N_{0}$; 40: **end if** 41: **for** i←0,1; **do** 42: **if** $m_{i}>m_{*}$ **then** 43: *T*.push(node.child[i]); 44: **else if** $m_{i}>0$ **then** 45: out.push($\left(S_{i},m_{i},p_{i}\right)$); 46: **end if** 47: **end for** 48: **end while**

### Appendix C.5. Adaptive Refinement: Variation 1

The original division algorithm of the adaptive EDOT (Algorithm A2) cuts a cell into two based on the balance of the averaged contribution of $W^{k}$ per discretization point between the divided cells. However, when the mass of μ is not distributed evenly in a cell to be cut, especially if it concentrates on a few small regions, the estimation of $W^{k}$ by the diameter of a cell becomes far greater than the actual one, thereby resulting in assigning much more discretizaiton points to a cell (Figure A1). Thus, we develop the division algorithm of Variation 1 to elevate the performance in this situation by estimating the Wasserstein distance using an occupied volume of a set of sample points (usually the same sample points we used in Algorithm A2): Given a resolution R>0 (the upper bound of a cell’s volume can be taken as $\mathrm{Vol}\left(X\right)/N_{0}$, with $N_{0}$ as the size of sample points), we keep cutting the region *X* until each cell is either of a volume smaller than *R* or contains no sample points; then, we call the total volume of those nonempty cells by the occupied volume $V_{Occ}\left(X\right)$. Similar definition applies to each cell $X_{i}$.

After having the occupied volume of each cell, we may proceed to assign a number of discretization points to each cell. The only improvement of division algorithm Variation 1 in this part is on the Wasserstein estimation step (line 12 and 13 in Algorithm A2), where the estimated Wasserstein $W^{k}$ of cell *i* is changed from $\mu \left(X_{i}\right)\cdot m_{i}^{-1/d}\cdot {\left(\mathrm{diam}X_{i}\right)}^{k}$ to $\mu \left(X_{i}\right)\cdot m_{i}^{-1/d}\cdot {\left(V_{\mathrm{Occ}}\left(X_{i}\right)\right)}^{k/d}$.

It is considered that the algorithm assigning the discretization size depends on the estimation of the Wasserstein distance; however, this estimation in Variation 1 requires the occupied volume, which is calculated from leaf to root, meaning that the binary tree for occupied volume has to be built before starting Algorithm A2 with a modified estimation of $W^{k}$. Fortunately, as the cutting points in this step have to coincide with the occupied-volume-calculation step, and the sample points belonging to each cell are both needed, we may save the sample points partition in the binary tree building for occupied volume and reuse them in discretization size assigning. Therefore, the discretization size assigning step works as a tree traversal (on a subtree with the same root, which is defined by the stopping conditions in depth along each path) of the binary tree built for occupied volume calculation.

Therefore, the time complexity for the occupied volume calculation is again $O(N_{0}\log N_{0})$, as the algorithm works in the same way as Quicksort again, and the time complexity for the rest (assigning discretization sizes) is O(m) as traversal on a tree of, at most, *m* leaves (*m* discretization points in total).

For space complexity, it is still $O(N_{0}+m)$, since after the calculation of occupied volume, the rest is only adding constant size decorations onto the subtree with, at most, *m* leaves mentioned above.

### Appendix C.6. Adaptive Refinement: Variation 2

The “cutting in the middle” method is easy to implement and guarantee the volume decreasing while going deeper in the tree (so the depth of getting under the resolution is guaranteed). However, it is also too rigid to fit the natural gaps of the original distribution, which may critically affect the optimal location of discretization points.

Our Variation 2 is on the dimension of redefining the cutting points from midpoints along the corresponding axis to the most sparse points. The sparsity is calculated by the moving window method along an axis/component of the *d*-coordinates; by applying the moving window method, we may have to sort the data points every time (since at each node, the sorting axis/component may be different). Since we still want to control the depth of the tree, a correction must be added to avoid the cutting point from locating too close to the boundaries (usually, the function $-\frac{C}{{\left(x-a\right)}^{k}{\left(x-b\right)}^{k}}$ with *a* and *b* the boundaries and C>0 as a constant). One influence is that now each cell’s volume (not the occupied volume) has to be calculated using the rectangular boundaries instead of being indicated only from its depth as before.

Thus, the influence on the time complexity is the following: 1. Changing the tree-building step to $N_{0}^{2}{\left(\log N_{0}\right)}^{2}$ in the average case, $N_{0}^{4}$ in worst case (if Quicksort is applied) on each node’s moving-window method), and 2. Introducing a $O(N_{0})$ for calculating the volume on each node in the binary tree. Furthermore, it introduces, at most, $O(N_{0})$ additional space complexity, since each cell’s volume has to be stored instead of being calculated directly from the depth.

Variation 2 can be applied together with Variation 1, since they are aiming at different parts of the algorithm. An example is shown in Figure A6.

## Appendix D. Empirical Parts

### Appendix D.1. Estimate $W_{k,\zeta }^{k}$: Richardson Extrapolation and Others

In the analysis, we may need $W_{k,\zeta }^{k}\left(\mu ,\mu_{m}\right)$ to compare how discretization methods behave. However, when the μ is not discrete, we are generally not able to obtain the analytical solution to the Wasserstein distance.

In certain cases, including all examples this paper contains, the Wasserstein can be estimated by finite samples (with a large size). According to [26], for μ∈P(X) in our setup (a probability measure on a compact Polish space with Borel algebra) and with $g=d_{X}^{k}\in C\left(X^{2}\right)$ being a continuous function, the Online Sinkhorn method can be used to estimate $W_{k,\zeta }^{k}$. The Online Sinkhorn needs a large number of samples for μ (in batch) to be accurate.

In our paper, as *X* are compact subsets in $R^{n}$, and μ has a continuous probability density function, we may use the Richardson Extrapolation method to estimate the Wasserstein distance between μ and $\mu_{m}$, which may require fewer samples and fewer computations (the Sinkhorn twice with different sizes).

Our examples are on intervals or rectangles, in which two grids $\Lambda_{1}$ of *N* points and $\Lambda_{2}$ of rN points (*N* and rN are both integers) can be constructed naturally for each. With μ determined by a smooth probability density function ρ, let $\mu_{\left(N\right)}$ be the normalization of $\sum_{i=1}^{N}\rho \left(\Lambda_{i}\right)\delta_{\Lambda_{i}}$ (this may not be a probability distribution, so we use its normalization). From a continuity of ρ and the boundedness of the dual variables α and β, we can conclude that

$$\lim_{N\to \infty }W_{k,\zeta }^{k}\left(\mu_{\left(N\right)},\mu_{m}\right)=W_{k,\zeta }^{k}\left(\mu ,\mu_{m}\right).$$

Let $W_{k,\zeta }^{k}\left(\mu_{\left(N\right)},\mu_{m}\right)$ be a function of 1/N; to apply Richardson extrapolation, we need the exponent of the lowest term of 1/N in the expansion $W_{k,\zeta }^{k}\left(\mu_{\left(N\right)},\mu_{m}\right)=W^{*}+O\left(1/N^{h}\right)+O\left({\left(1/N\right)}^{h+1}\right)$, where $W^{*}=W_{k,\zeta }^{k}\left(\mu ,\mu_{m}\right)$.

Consider that

$$\left|W_{k,\zeta }^{k}\left(\mu ,\mu_{m}\right)-W_{k,\zeta }^{k}\left(\mu_{\left(N\right)},\mu_{m}\right)\right|\leq W_{k,\zeta }^{k}\left(\mu ,\mu_{\left(N\right)}\right).$$

Since $W_{k,\zeta }\left(\mu_{\left(N\right)},\mu \right)\propto N^{-1/d}$, we may conclude that h=k/d, where *d* is the dimension of *X*. Figure A2 shows an empirical example in a d=1 and k=2 situation.

### Appendix D.2. Example: The Sphere

The CW complex structure of the unit sphere $S^{2}$ is constructed as follows: let (1,0,0), the point on the equator, be the only dimension-0 structure, and let the equator be the dimension-1 structure (line segment [0,1] attached to the dimension-0 structure by identifying both end points to the only point (1,0,0)). The dimension-2 structure is the union of two unit discs, which is identified to the south/north hemisphere of $S^{2}$ by stereographic projection:

(A33) $$\pi_{\pm N}:\left(X,Y\right)\to \frac{1}{1+X^{2}+Y^{2}}\left(2X,2Y,\pm (X^{2}+Y^{2}-1)\right)$$

with respect to the north/south pole.

#### Spherical Geometry

The spherical geometry is the Riemannian manifold structure induced by embedding onto the unit sphere in $R^{3}$.

The geodesic between two points is the shorter arc along the great circle determined by the two points. In their $R^{3}$ coordinates, $d_{S^{2}}\left(x,y\right)=\arccos\left(\langle x,y\rangle \right)$. Composed with stereographic projections, the distance in terms of CW complex coordinates can be calculated (and be differentiated).

The gradient about y (or its CW coordinate) can be calculated via the above formulas. In practice, the only problem is when x=±y function arccos at ±1 is singular. From the symmetry of sphere on the rotation along axis x, the derivatives of distance along all directions are the same. Therefore, we may choose the radial direction on the CW coordinate (unit disc). Furthermore, the differentiations are primary to calculate.

### Appendix D.3. A Note on the Implementation of SGD with Momentum

There is a slight difference between our implementation of the SGD and the algorithm provided in the paper. In the implementation, we give two different learning rates to the positions ($y_{i}$s) and the weights ($w_{i}$s), as moving along positions is usually observed much slower than moving along weights. Empirically, we make the learning rates on the positions be exactly three times the learning rates on the weights at each SGD iteration. With this change, the convergence is faster, but we do not have a theory or empirical evidence to show that a fixed ratio of three is the best choice.

Implementing and testing the Newton’s method (hybrid with SGD) and other improved SGD methods could be good problems to work on.

### Appendix D.4. An Example on Transference Plan with Adaptive EDOT

We now illustrate the performance of the adaptive EDOT on a 2D optimal transport task. Let $X=Y={\left[0,1\right]}^{2}$, $c=d_{X}$ be the Euclidean distance, $g=d_{X}^{2}$, and the marginal μ, ν be truncated normal (mixtures), where μ has only two components and ν has five components. Figure A4 plots the McCann Interpolation of the OT plan between μ and ν (shown in red dots) and its discrete approximations (weights are color coded) with m=n=30. With m=n=10, the adaptive EDOT results were as follows: $W_{k,\zeta }^{k}\left(\mu ,\mu_{10}\right)=1.33\times 10^{-2}$, $W_{k,\zeta }^{k}\left(\nu ,\nu_{10}\right)=1.30\times 10^{-2}$, and $W_{k,\zeta }^{k}\left(\gamma ,\gamma_{10,10}\right)=2.71\times 10^{-2}$. With m=n=30, the adaptive EDOT results were as follows: $W_{k,\zeta }^{k}\left(\mu ,\mu_{30}\right)=9.62\times 10^{-3}$, $W_{k,\zeta }^{k}\left(\nu ,\nu_{30}\right)=9.18\times 10^{-3}$, and $W_{k,\zeta }^{k}\left(\gamma ,\gamma_{30,30}\right)=1.516\times 10^{-2}$. The naive sampling results were as follows: $W_{k,\zeta }^{k}\left(\mu ,\mu_{30}^{\prime }\right)=1.75\times 10^{-2}$, $W_{k,\zeta }^{k}\left(\nu ,\nu_{30}^{\prime }\right)=1.58\times 10^{-2}$, and $W_{k,\zeta }^{k}\left(\gamma ,\gamma_{30,30}^{\prime }\right)=3.95\times 10^{-2}$. The adaptive EDOT approximated the quality of 900 naive samples with only 100 points on a four-dimensional transference plan.

### Appendix D.5. Example: Swiss Roll

In this case, the underlying space $X_{\mathrm{swiss}}$ is a the Swiss Roll, which is a 2D rectangular strip embedded in $R^{3}$: (θ,z)↦(θ,θ,z) in cylindrical coordinates. $\mu_{\mathrm{swiss}}$ is a truncated normal mixture on a (θ,z)-plane. Samples $S_{\mathrm{swiss}}∼\mu_{\mathrm{swiss}}$ over $X_{\mathrm{swiss}}$ are shown in Figure A5 (left) embedded in 3D and in Figure A6a as isometric into $R^{2}$.

By following the Euclidean metric in $R^{3}$, Figure A5 (right) plots the EDOT solution $\mu_{m}$ through adaptive cell refinement (Algorithm A2) with m=50. The resulting cell structure is shown as colored boxes. The corresponding partition of $S_{\mathrm{swiss}}$ is shown on Figure A6a, with samples contained in a cell marked by the same color. According to Figure A5 (right), the points in $\mu_{m}$ were mainly located on the strip, with only one point off in the most sparse cell (yellow cell located in the bottom in the figure).

On the other hand, consider the metric on $X_{\mathrm{swiss}}$ induced by the isometry from the Swiss Roll as a manifold to a strip on $R^{2}$. A more intrinsic discretization of $\mu_{\mathrm{swiss}}$ can be obtained by applying the EDOT through a refinement on the coordinate space—the (2D) strip. The partition of $S_{\mathrm{swiss}}$ is shown on Figure A6b, and the resulting discretization $\mu_{50}$ is shown in Figure A6c. Notice that all 50 points were located on the (locally) high density region of the Swiss Roll. We observe from Figure A6a,b that the 3D partition pulled disconnected and intrinsically remote regions together, while the 2D partition maintained the intrinsic structure.

### Appendix D.6. Example: Figure Densities

We used OpenCV to process the figures. The cat figure is a gray-scale figure of size 180×180. Variation 1 was used in cutting the figure into pieces, since the figure contains some sparse regions on which the original division algorithm does not work well.

For the colored figures (Starry Night and Girl with a Pearl Earring) in Figure 1, we process the three channels independently, then plotted the colored dots, and finally combined them as corresponding channels in a colored file. In the reconstruction of Starry Night, we made the size of the colored dots of same size with a modified color value according to the weights. Furthermore, for Girl with a Pearl Earring, we used pure color ((255,0,0) as red, etc.) and changed the size of the dots (with an area proportional to the weights).

### Appendix D.7. Example: Simple Barycenter Problems

The EDOT in simple form (no divide and conquer) can solve barycenter problems. The idea is simple: the gradient of a sum of functions is the sum of gradients of each function. Thus, to find the discrete barycenter of size *m* for several distributions $\mu_{i}$, we take the objective to be the sum of Wasserstein distances (raised to power *k* for rationality), whose gradient-to-target distribution is the sum of gradients between the discretization and each target distribution. This method only works for the simple EDOT, since there is no locality in barycenter problems. After a division, the weights of each target distribution in each cell of the partition may be different, so there is inter-cell transport in the optimal transport plan, which the current algorithm cannot deal with.

We can see in Figure A7 that the simple EDOT-Equal (no divide and conquer) achieved similar results as the non-regularized discretization in [18], whereas the EDOT produced a better approximation of the barycenter by taking advantage of changing weights freely.
